# Revisiting regulatory T cells as modulators of innate immune response and inflammatory diseases

**DOI:** 10.3389/fimmu.2023.1287465

**Published:** 2023-10-20

**Authors:** Qifeng Ou, Rachael Power, Matthew D. Griffin

**Affiliations:** ^1^ Regenerative Medicine Institute (REMEDI) at CÚRAM SFI Research Centre for Medical Devices, School of Medicine, College of Medicine, Nursing and Health Sciences, University of Galway, Galway, Ireland; ^2^ Nephrology Department, Galway University Hospitals, Saolta University Healthcare Group, Galway, Ireland

**Keywords:** regulatory T cells (Treg), innate immune response, macrophages, dendritic cells, immune regulation, neutrophils, inflammatory diseases, cell therapies

## Abstract

Regulatory T cells (Treg) are known to be critical for the maintenance of immune homeostasis by suppressing the activation of auto- or allo-reactive effector T cells through a diverse repertoire of molecular mechanisms. Accordingly, therapeutic strategies aimed at enhancing Treg numbers or potency in the setting of autoimmunity and allogeneic transplants have been energetically pursued and are beginning to yield some encouraging outcomes in early phase clinical trials. Less well recognized from a translational perspective, however, has been the mounting body of evidence that Treg directly modulate most aspects of innate immune response under a range of different acute and chronic disease conditions. Recognizing this aspect of Treg immune modulatory function provides a bridge for the application of Treg-based therapies to common medical conditions in which organ and tissue damage is mediated primarily by inflammation involving myeloid cells (mononuclear phagocytes, granulocytes) and innate lymphocytes (NK cells, NKT cells, γδ T cells and ILCs). In this review, we comprehensively summarize pre-clinical and human research that has revealed diverse modulatory effects of Treg and specific Treg subpopulations on the range of innate immune cell types. In each case, we emphasize the key mechanistic insights and the evidence that Treg interactions with innate immune effectors can have significant impacts on disease severity or treatment. Finally, we discuss the opportunities and challenges that exist for the application of Treg-based therapeutic interventions to three globally impactful, inflammatory conditions: type 2 diabetes and its end-organ complications, ischemia reperfusion injury and atherosclerosis.

## Introduction

Regulatory T cells (Treg), classically identified in humans and other species as CD4^+^/Foxp3^+^ lymphocytes, comprise only 5-10% of total CD4^+^ T cells, but are essential for balancing immune system activity throughout life. The immunosuppressive and immune modulatory functions of Treg have been unequivocally established in the context of adaptive immunity and have become fundamental to our understanding of autoimmunity, transplant rejection, tumor antigen-specific immune responses and the distinction between harmful and commensal microorganisms (1). Less well appreciated has been the accumulating research evidence that Treg directly modulate activity of the innate immune system through cross-talk with mononuclear phagocytes (monocytes/macrophages), granulocytes (neutrophils), dendritic cells (DC) and innate lymphocytes (NK cells, NKT cells, γδ T cells and other innate lymphocytic cells (ILC)) (2). The impacts of innate immune cell interactions with Treg may include modifications to cell survival, proliferation, cytokine production, phagocytosis, cytotoxicity and other effector functions. Although Treg-mediated suppressive effects on innate immune cells can be implicated in their regulation of antigen-specific T- and B-cell responses, they may also be more broadly relevant to immune-mediated inflammatory diseases (IMID) and disorders of tissue homeostasis, such as diabetes, atherosclerosis, Parkinson’s disease, wound healing and aging (3). This suggests that a deeper understanding of the Treg phenotypic and functional properties that underlie their modulation of innate immune responses could open new translational pathways for the burgeoning field of regulatory immune cell therapies (4).

In this review, we aim to summarize current knowledge of the direct modulatory effects of Treg on diverse innate immune cells types with an emphasis on mechanisms and clinically-meaningful contexts in humans as well as experimental animals. We also discuss the relevance of these insights to common human diseases and health challenges for which non-autoimmune inflammation is a key component and reflect on the prospects for Treg-based therapies to be applied to their treatment.

Although the majority of the studies described here focus on the canonical CD4^+^CD25^+^CD127^-^ Foxp3^+^ Treg phenotype, it is important to acknowledge that there is considerable heterogeneity in regard to Treg subtypes and functionality. Most clearly, CD4^+^ Treg may be divided into “natural” or thymic-derived (tTreg), peripherally-derived (pTreg) and *in-vitro* induced Treg (iTreg). In addition, however, sub-populations defined on the basis of specific surface markers, transcription factors, functional properties or disease associations have been described, including type 1 regulatory T cells (Tr1), Th3 cells, FoxA1^+^ Treg (5), CD39^+^ Treg, CD8^+^ Treg, Treg-of-B and others (6, 7). Where relevant, therefore, we highlight Treg subpopulations that have been described to regulate specific aspects of innate immune response.

## Regulatory T cell interactions with mononuclear phagocytes and granulocytes

### Mononuclear phagocytes

**Figure 1:**
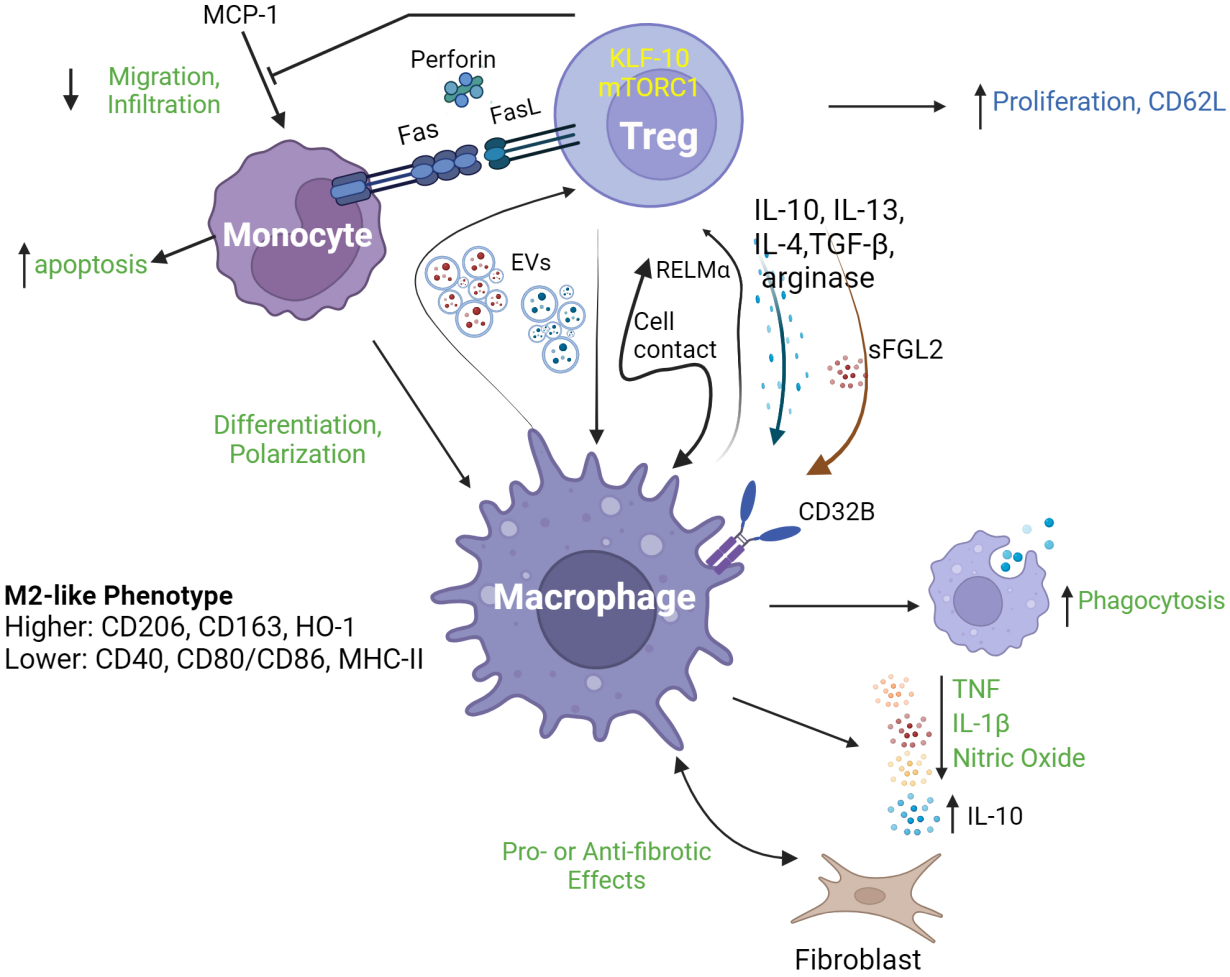
*Regulatory T cell interactions with mononuclear phagocytes.* Regulatory T cells (Treg) interact with mononuclear phagocytes including monocytes and macrophages, through a range of mechanisms leading to a diverse array of consequences. As shown in the upper part of the figure, Treg modulate several aspects of monocyte behavior, including their survival (through death receptor and cytolytic pathways), migration/infiltration (through inhibition of MCP-1) and differentiation into M2-type macrophages. Such functions may be dependent on specific Treg transcriptional (KLF-10) and signaling (mTORC1) programs. As depicted in the lower part of the figure, Treg and macrophages may engage in complex cross-talk including release/expression of signature modulatory cytokines (IL-10, IL-13, IL-4 and TFG-β), arginase, RELMα and sFGL2; and exchange of extracellular vesicles (EVs). Outcomes of this cross-talk may include enhanced macrophage phagocytosis, release of pro-inflammatory mediators (TNF, IL-1β and nitric oxide) and modulation of the communication between macrophages and fibroblasts, as well as increased Treg proliferation and expression of CD62L. EVs, extracellular vesicles; RELMa, resistin-like molecule α; MCP-1, monocyte chemoattractant protein-1; KLF-10, Krüppel-like factor 10; mTORC1, mechanistic target of rapamycin complex 1; sFGL2, soluble fibrinogen-like protein 2; HO-1, haemoxygenase 1.

Accumulating evidence supports the existence of a biologically significant role for Treg in regulating monocyte- and macrophage-mediated inflammation in diverse contexts. For example, the co-occurrence of decreased Treg proportions and increased monocyte inflammatory mRNA expression was identified in the blood of adults with major depressive disorders (8). In contrast, in patients with acute HIV-1 infection, increased proportions of Foxp3^+^Helios^+^CD45RA^+^ Treg were reported along with decreased frequencies of CD14^++^CD16^+^ (intermediate) monocytes and increased proportions of PD-1^+^ cells among both CD14^++^CD16^+^ and CD14^+^CD16^++^ (non-classical) monocytes (9). In scurfy mice, an animal model in which Foxp3 mutation results in profound Treg deficiency, increased myelopoiesis and monocyte homing receptor expression, phagocytic activity and cytokine production occurs (10). Recently, Hand et al. reported that diurnal variations of Treg numbers occur within joints of mice and that depletion of Treg resulted in increased joint inflammation at night in association with enhanced production of IL-1β by Ly6C^hi^ monocytes (11). These observations of inverse relationships between Treg and monocyte activities indicate a potential role of Treg in directly regulating the inflammatory profile of mononuclear phagocytes. In both ex vivo human cell experiments and *in vivo* animal models, Treg have been demonstrated to promote monocyte differentiation toward alternatively activated/anti-inflammatory (M2) macrophage phenotypes characterized by high phagocytic activity and expression of related marker proteins (CD206, CD163, and heme oxygenase-1); low antigen-presentation capacity (CD40, CD80/86, and class II MHC); increased secretion of anti-inflammatory cytokines (IL-10) and decreased production of pro-inflammatory molecules (TNF, IL-6, nitric oxide (NO) and reactive oxidative species (ROS)). Such influences of Treg on mononuclear phagocyte differentiation have been reported to be both cell contact- (12, 13) and cytokine-dependent. In the case of soluble mediators, Treg IL-10 (14), IL-13 (15), TGF-β, IL-4 (16), arginase (17), and soluble fibrinogen-like protein 2 (18) have all been shown to play a role in various experimental settings. The transcription factor Krüppel-like factor 10 (KLF10) (19) and the signaling pathway mediated by mammalian target of rapamycin complex 1 (mTORC1) have also been reported to be necessary for Treg suppression of macrophage-mediated inflammation (20). Recently, exosome-based cross-talk between Treg and macrophages has also been documented. In a mouse model of acute myocardial infarction, Hu et al. reported that infusion of Treg-derived exosomes reduced infarct size and improved cardiac function by promoting macrophage polarized towards M2 phenotypes in myocardial tissue (21). In a reverse interaction, Zhou et al. demonstrated that macrophage-derived exosomes transferred miR-29a-3p and miR-21-5p into CD4^+^ T cells to promote their differentiation into Treg, in a mouse tumor microenvironment (22). Furthermore, Treg-supported M2 macrophages can induce CD4^+^CD25^-^ T cell to acquire a suppressive phenotype, forming a positive feedback loop in immune regulation (23). Mechanistically, macrophage promotion of Treg induction, proliferation and migration has been linked to an IL-10 and TGF-β-secreting (M2c) phenotype, to the secretion of resistin-like molecule (Relm)-α and to the induction of CD62L on Treg (24–26). **Figure 1** illustrates a range of reported interactions between Treg and mononuclear phagocytes along with their key mediators and outcomes.

From a therapeutic perspective, Romano et al. have reported that ex vivo expanded human Treg may be more potent in suppressing monocyte activation than freshly-isolated Treg. In this study, the effects of ex vivo expanded Treg on activated human monocytes were manifested as reduction of NF-κB signaling, a higher proportion of CD14^+^CD206^+^CD163^+^CD86^−^ (M2-like) cells and compromised potency to induce Th17 cell responses (27). Using a CD28 super-antagonist, Cai et al. recently showed that *in vivo*-expanded endogenous mouse Treg adoptively transferred to diabetic animals undergoing ischemic stroke induced an increase in CD206^+^ M2 microglia/macrophages in ischemia tissue detected by real-time visualization using an optical imaging probe (28).

In addition to the initiation and resolution of inflammation, monocytes and tissue macrophages also play an important role in the development of post-inflammatory fibrosis through direct effects or via interactions with fibroblasts. In the short-term, the pro-fibrotic effects of alternatively-activated macrophages are beneficial to wound healing and tissue repair, whereas their prolonged pro-fibrotic activity contributes to the pathogenesis of a range of disease processes (29). For example, fibrosis of lung (30) and liver (31) as well as chronic kidney disease (32) and systemic sclerosis (33), have all been reported to be linked with macrophage-induced fibrosis. Currently, the potential influence of Treg interactions with mononuclear phagocytes on physiological and pathological tissue fibrosis is not well understood. Of interest, Song et al. recently reported that adoptively transferred Treg accelerated tissue repair and kidney function recovery in mice following reversal of unilateral ureteral obstruction in association with higher intra-renal proportions of M2 macrophages (34). Nonetheless, the concept of Treg ameliorating fibrosis through M2-like polarization of macrophages is not entirely in accordance with current understanding of M1/M2 balance in disease-associated fibrosis. For example, in animal models of diabetic kidney disease, pharmacological interventions which alleviated renal fibrosis and preserved kidney function have variously been reported to promote M1 (35) or M2 (36) macrophage phenotypes. Furthermore, Kim et al. demonstrated that M2c macrophages, induced with IL-10 and TGF-β from mouse bone marrow-derived macrophages, exacerbated renal fibrosis following acute kidney injury (32). Bhandari et al. also reported that a subset of human macrophages capable of activating fibroblasts and promoting fibrosis exhibited both M1- and M2-associated surface marker expression and cytokine production in the setting of systemic sclerosis (33), while Tan-Garcia et al. observed enrichment of TNF-secreting, CD206^+^ macrophages in fibrotic liver of hepatocellular carcinoma (31). Such results indicate that the classical dichotomy of macrophage M1/M2 polarization frequently fails to explain the various roles of macrophages in fibro-inflammatory disease and, by extrapolation, raise the possibility that Treg modulation of mononuclear phagocyte activation and differentiation could also exacerbate disease in some settings (37). In keeping with this, pro-fibrotic Treg subtypes have been identified as participating in ventricular remodeling after heart failure (38) and in lymphatic tissue fibrosis after HIV infection (39). In addition, adoptively transferred Treg have been reported to worsen the pro-fibrotic environment in bleomycin-induced lung injury models (40). In non-fibrotic diseases also, the effects of Treg on macrophage phenotype may be more ambiguous than simply modulating from M1 to M2. For example, Treg have been shown to decrease macrophage infiltration in lesional skin, resulting in reduced disease progression in a model of psoriasis (41). However, the macrophage phenotypes that predominate in psoriasis progression may include CD68^+^iNOS^+^ (M1), CD206^+^ (M2), or phenotypes distinct from M1/M2 (42). These studies highlight the complexity of predicting the outcome of Treg/mononuclear phagocyte cross-talk in different contexts as well as the need for carefully designed pre-clinical studies to guide the development of Treg-based therapies for non-autoimmune acute and chronic inflammatory diseases. Further investigation of the phenotypes, origin, migration and pro/anti-inflammatory function of monocytes and macrophages will help to develop a deeper understanding of how Treg modulate their activity in specific disease settings.

Regulatory T cells also express multiple mediators of programmed cell death and may suppress inflammation through induction of apoptosis in mononuclear phagocytes (**Figure 1**). While the killing of CD4^+^ T cells by Treg is thought to be based on TRAIL/TRAILR and galectin pathways (43), the mechanisms underlying monocyte killing by Treg is controversial. Venet et al. reported that human Treg induced CD14 down-regulation – a marker of apoptosis - in LPS-activated monocytes through a FasL/Fas interaction (44). In contrast, however, Jagger et al. concluded from co-culture experiments that FasL/Fas-mediated killing of activated human monocytes is mediated by effector T cells (Teff) rather than Treg, and that the effects of Treg were predominantly suppressive rather than pro-apoptotic (45). Nevertheless, Grossman et al. have also reported Treg to be capable of inducing apoptosis via perforin in multiple cell types, including CD14^+^ monocytes (46). Thus, the significance of Treg-mediated killing in modulating mononuclear phagocyte-associated inflammation requires further investigations.

Effects of Treg on phagocytosis in the context of inflammatory disease have also been reported (**Figure 1**). In atherosclerosis, for instance, phagocytosis by macrophages contributes to the resolution of arterial wall plaques and, in this context, Sharma et al. have recently demonstrated, in mice, that Treg prime resident macrophages to be phagocytic and pro-resolving. Mechanistically, this effect was mediated by Treg-secreted IL-10 and macrophage-derived IL-13 (47). Finally, there is evidence that resident Treg have specialized functions for local regulation of mononuclear phagocyte activity in inflammation-sensitive tissues. For example, Xie et al., reported that cerebral, but not splenic, Treg modulated the phenotype (upregulated CD163, downregulated RTIB) and cytokine production (downregulated TNF, IL-1β, and IL-6) of resident macrophages (microglial cells) in an IL-10-dependent manner, to maintain immune homeostasis in the central neuron system (14).

### Granulocytes

In comparison to mononuclear phagocytes, there has been relatively limited investigation of Treg interactions with granulocytes. Nonetheless, it has been proposed that Treg can mediate anti-inflammatory and pro-tolerogenic effects through their influences on neutrophils. As described by Lewkowicz et al., Treg activated by LPS or CD3/CD28 ligation induced expression of a range immune suppressive pathways in neutrophils through different mediators in addition to promoting their apoptosis (48). In a subsequent study, these authors showed that human neutrophils co-cultured with activated Treg, had increased expression of anti-inflammatory molecules (IL-10, TGF-β1, IDO and HO-1). Upregulation of IDO was dependent on Treg expression of CTLA-4, although the corresponding target on neutrophils was not clear (49). In a mouse model of sepsis complemented by human co-culture experiments, Okeke et al., demonstrated that a PI3Kσ-dependent Treg function limited severe inflammation by promoting neutrophil apoptosis (50). Adoptively transferred Treg have also been shown to improve intestinal barrier function in association with decreased frequency of neutrophils in the intestinal tissue in the setting of heatstroke (51). In a meticulous study of mouse autoimmune hepatitis, Umeshappa et al. observed that autoantigen-specific Tr1 cells together with B regulatory cells (Breg), orchestrated neutrophil recruitment to the liver and reprogrammed them to myeloid-derived suppressor cell (MDSC) subtypes via GM-CSF, IL-10, and TGF-β. These regulatory neutrophils subsequently protected liver tissue from autoimmune injury via cathelin-related anti-microbial peptide (CRAMP) (52). Of interest, human Treg have been shown to recruit neutrophils *in vitro* by secretion of CXCL8 (53). Furthermore, neutrophils have been found to be spatially distributed closer to Foxp3^+^ Treg and to potentially modulate CD4^+^ T cell differentiation via PD-L1/PD-1 interactions (54). Less well understood, however, is the relationship of Treg with NETosis, a process by which neutrophil extracellular traps (NETs) are extruded by neutrophils prior to cell death. A positive correlation between Treg with high-risk NETosis score was identified in ANCA glomerulonephritis (55). However, it has also been reported that Treg tend to have no impact on NETosis of polymorphonuclear neutrophils stimulated by either immune complex or PMA (56). Inversely, in diverse animal model settings, NETs have been shown to promote Treg differentiation from naive CD4^+^ T cells and to be supportive of Treg suppression potency (57). Nonetheless, NETs have also been reported to promote differentiation of naïve CD4^+^ T cells towards Th17-like phenotype either *in vitro* or *in vivo (* 58).

**Figure 2:**
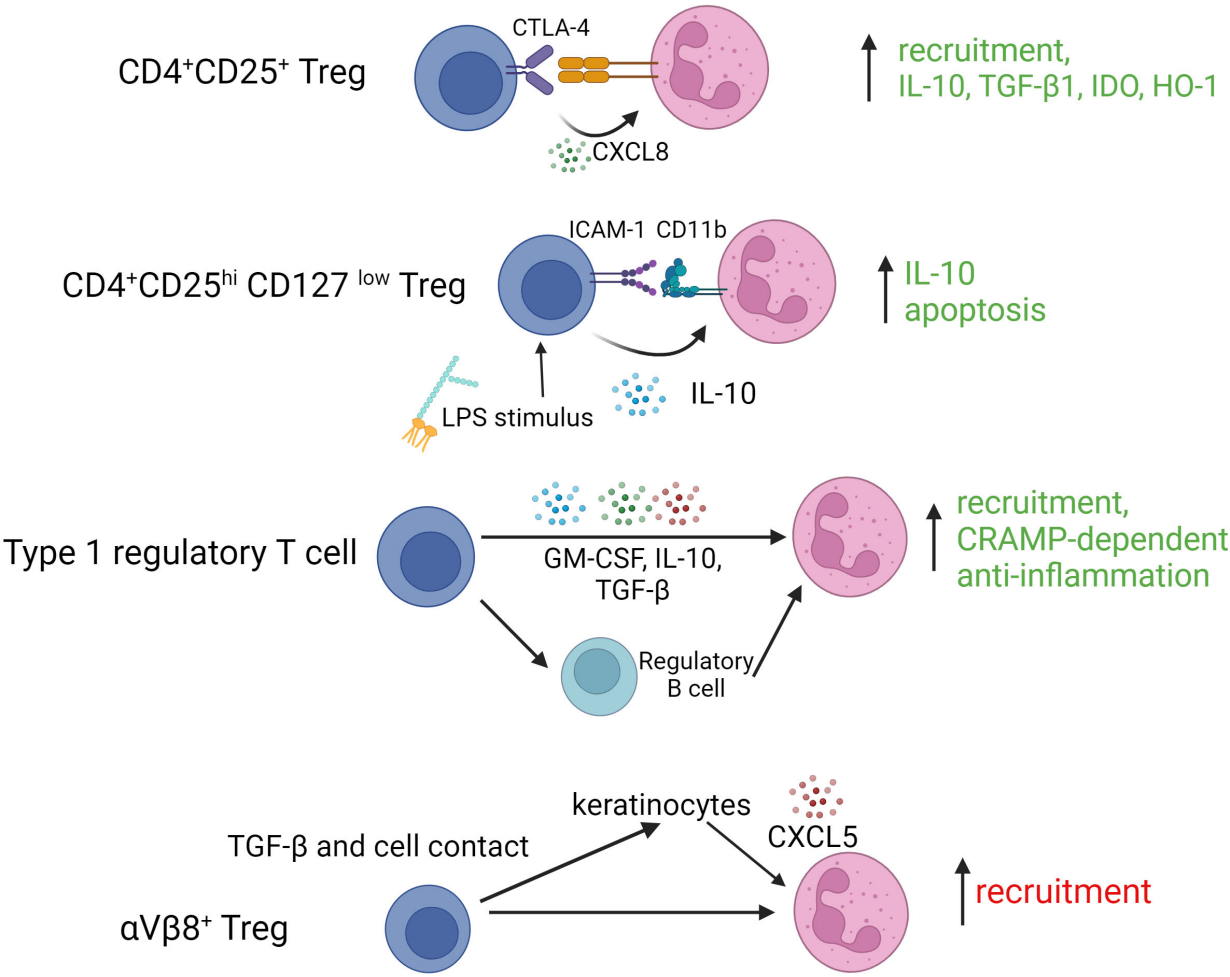
*Interactions of various regulatory T cell subtypes with granulocytes*. TGF-β1, transforming growth factor beta 1; IDO, indoleamine-2,3-dioxygenase; HO-1, hemoxygenase 1; ICAM-1, intercellular adhesion molecule 1; LPS, lipopolysaccharide; GM-CSF, granulocyte macrophage colony stimulating factor; αVβ8, integrin alphaVbeta8.

As is clear from these studies, different Treg subtypes are capable of complex cross-talk with neutrophils during acute and chronic immune/inflammatory responses. How such interactions influence disease outcomes or could be exploited in therapeutic contexts remains unclear and it should be acknowledged that some of the published work in this area indicates a potential for detrimental effects. For example, Treg in skin expressing the integrin αVβ8 use latent-activated TGF-β in a cell contact-dependent manner to induce CXCL5 expression by keratinocytes, recruiting neutrophils and, as a result, contributing to a delayed epithelial regeneration (59). **Figure 2** summarizes reported Treg granulocyte interactions along with their key mediators and functional outcomes.

Mast cells (MC), as a type of tissue-resident granulocytes, play a pro-inflammatory role in parasite infection and anaphylaxis in addition to mediating regulatory effects on innate and adaptive immune responses under other conditions. In the past two decades, it has become increasingly clear that Treg modulate MC activation and migration in different clinical settings and by numerous mechanisms. A prototypical mechanism is cell-cell contact based on OX40 (on MC) and OX40L (on Treg) resulting in downregulation of the Fyn/Gab2/PI3K pathway and suppressed degranulation of MC, halting allergic inflammation (60). Treg-derived TGF-β has been reported to prime MC IL-6 production, which facilitates clearance of neutrophils in innate lung inflammatory (61) – an effect that is dependent on membrane-bond rather than soluble TGF-β (62). Treg-derived TGF-β is also reported to function in counterpart with IL-4 to modulate MC activation by reciprocal downregulation of their receptors (63). Unlike TGF-β, Treg-secreted IL-9, in a soluble form, promotes MC migration to skin allografts to promote immune tolerance (64), or to draining lymph node to promote renal protection in the nephrotoxic serum nephritis model (65). However, one study has reported that Treg suppressed rather than enhanced IL-9 production, resulting in abrogation of IL-9-dependent MC degranulation and aggravation of intestinal nematode burden in S. ratti infected mice (66).

Mast cells, in turn, influence Treg phenotype and function in similar fashion to Treg cross talk with monocytes and neutrophils. For example, MC-derived IL-2 is indispensable for maintaining resident Treg/Teff ratio for skin homeostasis in chronic allergic dermatitis (67). Similar effects on Treg were also observed for MC-secreted TGF-β (68). In contrast, MC-derived histamine muted Treg suppressive potency on effector T cells by signaling through the H1 receptor *in vitro* (69). Mast cells, in particular stimulated by IL-33 (70), may also drive Treg into Th17-like phenotypes via OX40/OX40L-mediated cell contact in presence of MC-derived IL-6 (71). Overall, therefore, the interplay between Treg and MC is clearly complex with diverse potential outcomes, necessitating further, context-dependent research before it can be directly exploited for therapeutic purposes. A recent comprehensive review focused on therapeutic opportunities for cholangiopathies provides a more detailed delineation of Treg/MC cross talk (72).

## Regulatory T cell interactions with dendritic cells

Dendritic cells represent a key interface between innate and adaptive immunity, sensing microbial and other threats in tissues through pattern recognition and responding by producing soluble mediators of inflammation as well as by migrating to secondary lymphoid organs while processing and presenting foreign peptides to activate naïve antigen-specific CD4^+^ and CD8^+^ T cells (73). The interplay between DC and Treg has been extensively studied and Treg/DC cross-talk has been established to be an important pathway by which Treg support immune tolerance to self- and non-threatening foreign antigens (74). Although the focus of this review is on Treg effects on innate immunity, the complex mechanisms underlying Treg interactions with DC and their downstream influences on both innate and adaptive aspects of DC functions merit some description as they provide important insights into the potential for Treg-based therapies to be applied to a range IMID.

In this regard, both surface interactions and exchange of soluble mediators between Treg and DC are of significant interest. As potent antigen-presenting cells, activated DC abundantly express the B7 family surface proteins CD80 and CD86 which mediate co-stimulation for initialization of Teff activation by binding to the CD28 receptor (75). In contrast, Treg inherently express higher levels of CTLA-4 (CD152) which competes with CD28 for binding to CD80/CD86 and, thereby, limits DC-stimulated Teff responses. In addition to competing with CD28, however, Treg are also known to directly downregulate DC expression of CD80/CD86 through CTLA-4 (76). Of interest, one mechanism for this downregulation has been directly visualized as a physical process by which CD80/CD86 molecules are removed from the DC surface by Treg and internalized (trans-endocytosis) (77) or incorporated into the Treg surface membrane (trogocytosis) (78). Depletion of CD80 from the DC surface may lead to an increase of free PD-L1 surface expression, favoring co-inhibition of Teff activation (79). Thymic-derived Treg have also been shown to induce endocytosis of CD70 from the surface of DC, thereby inhibiting the CD70/CD27 pathway which is required for DC-primed Th1 responses (80). It is also noteworthy that the mechanisms and outcomes of these interactions could vary between secondary lymphoid tissues and inflamed organs such as autoimmune target organs or transplants. For example, Dai et al. recently reported in an allogeneic islet transplant model that, while Treg contacts with DCs and suppression of DCs/T effector interactions within the inflamed allograft were dependent on adenosine generated by the ecto-nucleotidase CD73, contacts between Treg and DCs within the spleen were independent of CD73 (81).

Soluble factors secreted by Treg, in particular IL-10 and TGF-β, also suppress DC surface expression of key proteins including HLA-DR, CD80/CD86 and CD40 as well as their production of pro-inflammatory cytokines such as TNF and IL-12 (82, 83). Another cytokine, IL-35, produced predominantly by thymic Treg, has been reported, in mice, to induce tolerogenic DC characterized by increased expression of CD11b and IL-10 and decreased MHC-II (84). Zhang et al., have demonstrated the combined roles of human Treg cell surface proteins (CTLA-4) and soluble factors (IL-10 and TGF-β) in inducing a type of tolerogenic DC from primary monocytes in the presence of T helper cells (85). Another mechanism for non-contact-dependent modulation of DC by Treg occurs through release and uptake of extracellular vesicles (EVs). For example, Tung et al. reported that murine CD4^+^Foxp3^+^Treg-derived EVs induced increased IL-10 and decreased IL-6 production in LPS-activated DCs, and this modulated cytokine production is in association with evidence of EV-mediated trafficking of miR-150-5p and miR-142-3p (86).

The question of whether Treg modulation of DC functions is dependent on recognition of DC-presented MHC II/peptide complexes by the Treg remains under investigation and is of particular importance to the clinical translation of Treg therapies for immune-mediated diseases without a well characterized auto- or allo-immune basis. In this regard, it is reasonable to hypothesize that antigen-specific Treg mediate more potent modulation of inflammatory processes than polyclonal Treg if DCs presenting their cognate antigens are present. Indeed, in the setting of experimental autoimmune (type 1) diabetes, antigen-specific Treg, based on a unique TCR repertoire capable of recognizing specific class II MHC-presented peptide antigens, have been clearly shown to prevent disease more potently than polyclonal Treg (87, 88). Furthermore, adoptively transferred or *in vivo*-induced antigen-specific Treg are considered to have enhanced potency to contribute to disease-specific immune regulation, consequently necessitating a lower dose of infused Treg and reducing the potential for systemic immunosuppression (89). In keeping with this, Akkaya et al. observed, in co-cultures, that mouse antigen-specific iTreg formed stable compact clusters around cognate antigen-pulsed DCs and stripped off MHC-II peptide complexes from the DC surface to compromise DC-induced activation of naïve antigen-specific Teff. This interaction was not present in co-cultures of polyclonal Treg with DC. The findings were replicated in *in vivo* experiments, further supporting the conclusion that Treg capture of MHC/peptide complexes from DC was antigen-specific (90). In a study by Liang et al., Treg expanded by culture with antigenic peptide-bearing DCs, subsequently suppressed DC maturation through Lag-3:MHC-II-based cell contact only in the presence of peptide (91). Of note, however, polyclonally expanded Treg were not used as controls in these studies. Furthermore, others, have provided evidence that Treg suppress DC antigen presenting potency in an MHC-independent manner. For example, Chen et al. and Yan et al, reported that polyclonal Treg suppress DC interaction with conventional T cells, by inducing changes in cytoskeletal polarization through stable integrin-mediated contact independent of antigen and MHC-II (92, 93). In the study of Yan et al., IL-2 conditional polyclonal Treg were shown to build stable connections with MHC-II-deficient DCs and such conditioned DCs were incapable of activating CD8^+^ T cells (93). Specific molecular interactions reported to be involved in the strong adhesion between Treg and DC include LFA-1/ICAM-1 (92, 94); integrin α4β1 (95) and Nrp1 (96), regardless of MHC restriction. Overall, more investigation is needed to characterize and validate the mechanisms underlying Treg/DC physical interactions and to exploit non-antigen-specific Treg modulation of DC in inflammatory diseases that lack an auto- or allo-immune component. More broadly, whether TCR/MHC peptide engagement is necessary for or enhances Treg suppression of the pro-inflammatory functions of other MHC II-expressing innate immune effectors such as monocytes/macrophages and granulocytes merits additional study.

**Figure 3:**
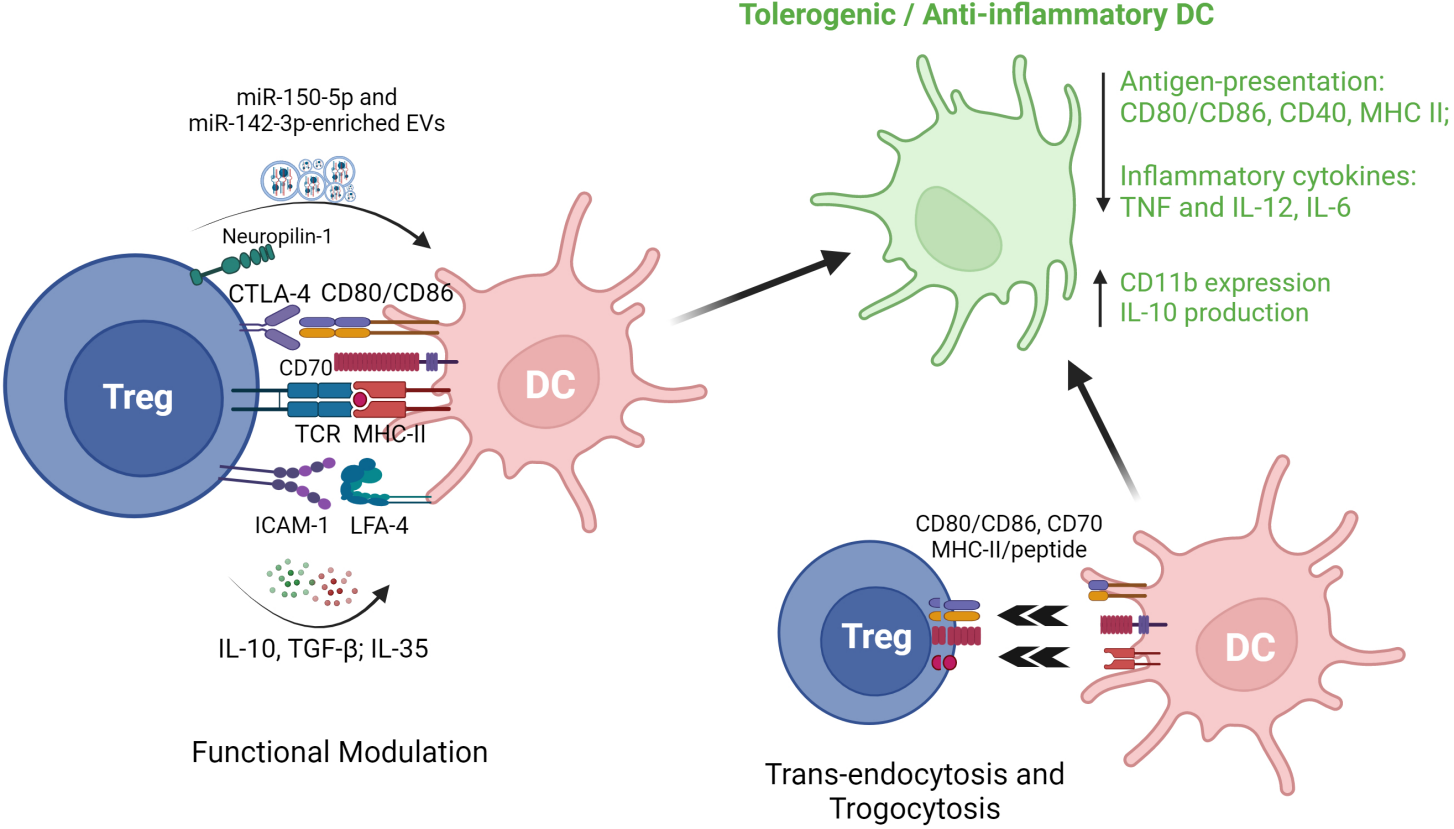
*Regulatory T cell interactions with dendritic cells*. EVs, extracellular vesicles; CTLA-4, cytotoxic T lymphocyte antigen 4; TCR, T cell receptor; MHC II, major histocompatibility complex II; ICAM-1, intercellular adhesion molecule 1; LFA-1, lymphocyte function-associated antigen 1; DC, dendritic cells; TGF-β1, transforming growth factor beta 1; TNF, tumor necrosis factor.

Besides canonical CD4^+^Foxp3^+^ Treg, regulation of DC by other types of Treg has also been documented. In a study involving mouse and human experiments, Liu et al. reported that FoxA1^+^ Treg with a deficiency in Foxp3 expression yet a higher expression of PD-L1, reduce IL-12 and IL-17 production by LPS-activated HLA-DR^+^ antigen presenting cells (APC) (5). Other studies have investigated the cross-talk between IL-10-secreting DC (DC-10) and Tr1 cells (97), demonstrating that co-culture of human DC-10 with CD4^+^ T cells results in the induction of antigen-specific Tr1 in the setting of autoimmune disease (98) and organ transplantation (99). Initially identified and purified as CD14^+^CD11c^+^CD83^+^ cells (100), DC-10 could also be generated from bead-selected CD14^+^ monocytes by culture in presence of GM-CSF and IL-4 (99). Recently, a better-defined DC-10 population (CD14^+^CD16^+^CD141^+^CD163^+^) has been purified from blood and shown to exhibit similar IL-10 production and Tr1 induction potency (101). Importantly, ex vivo generated Tr1 have been recognized as a promising anti-inflammatory cell therapy and may modulate DC to more tolerogenic phenotypes characterized by higher expression of ILT-3, ILT-4 and HLA-G. Induced Tr1 can also release granzyme B and perforin through CD2/CD58- and CD226/CD155-based cell contact, to induce cell death in myeloid cell-derived APC. Despite lack of Foxp3 expression, Tr1 express key Treg-associated receptors (e.g. CTLA-4, PD-1 and ICOS) and are capable of similar suppressive regulation of DC through cell-cell contact (102). A range of the reported mechanisms of Treg modulation of DC phenotype and function are summarized in **Figure 3**.

## Regulatory T cell interactions with innate or innate-like lymphocytes

### Natural killer cells

**Figure 4:**
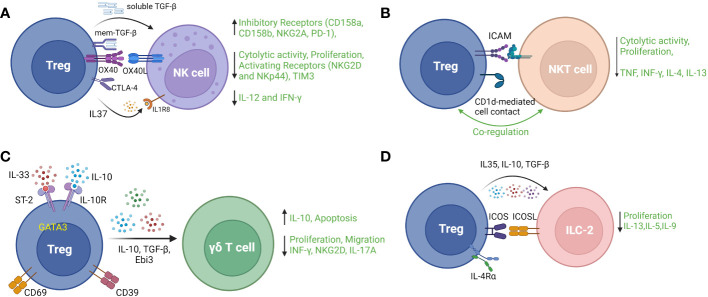
*Regulatory T cell interactions with four main types of innate or innate-like lymphocytes*: **(A)** Natural Killer (NK) cells, **(B)** Natural Killer T (NKT) cells, **(C)** Gamma delta (γδ) T cells **(D)** type 2 innate lymphoid cells (ILC-2). TGF-b – transforming growth factor beta 1; NKG2A, natural killer group 2A; PD-1, programmed cell death 1; NKG2D, natural killer group 2D; TIM3, T cell immunoglobulin and mucin domain-containing protein 3; NKp44, natural killer p44; IFNγ, interferon gamma; ICAM – intercellular adhesion molecule; TCR, T cell receptor; TNF, tumor necrosis factor; ST-2, suppression of tumorigenicity 2; IL-10R, interleukin 10 receptor; Ebi3, Epstein Barr virus-induced; ICOS, inducible T cell costimulatory; ICOSL, inducible T cell costimulatory ligand; IL-4Rα, interleukin 4 receptor alpha.

Natural killer cells, a subset of innate immune cells derived from lymphoid progenitors, play important roles in the control of viral infection and tumorigenesis in an MHC-independent manner. The effector functions of NK cells include cytolysis and induction of programmed cell death in target cells as well as production of pro-inflammatory cytokines. Numerous studies have provided evidence that Treg counteract NK cell effector functions by decreasing cytolytic activity and proliferation, downregulating activation receptors (NKG2D and NKp44), upregulating inhibitory receptors (CD158a, CD158b, and NKG2A) (103), and suppressing production of IL-12 and IFN-γ (104). Mechanistically, membrane-bound TGF-β on Treg has been shown to be an important mediator of Treg suppression of NK cells response in the setting of cancer (104). In a mouse model of hepatitis B, Treg-mediated inhibition of NK cell cytotoxicity was attenuated by blockade of OX-40L, identifying a role for the OX-40/OX-40L pathway in protecting against NK cell-mediated chronic liver injury in this infection (105). Intrahepatic Treg also suppressed NK cell degranulation and NKG2D expression in a CTLA-dependent manner, contributing to decreased liver fibrosis by regulating NK cell interactions with hepatic stellate cells (106). Secretion of IL-37 by Treg is reported to suppress NK cell proliferation, cytotoxicity and IFN-γ production through its receptor IL-1R8 in association with NK cell downregulation of TIM-3 and upregulation of PD-1 (107). In a mouse model of autoimmune diabetes, an indirect mechanism of suppression of NK cells by Treg has been described by which Treg consumed IL-2 produced by islet-infiltrating CD4^+^ cells, thereby curbing the localized expansion of NK cells (108). A recent study also suggested that IL-10^+^ Treg suppress cytolytic activity of NK cells in acute retroviral infection. In this study, Littwitz-Salomon et al. showed that Treg were activated with increased expression of ICOS, IL-10 and TGF-β during the early, acute phase of Friend Retrovirus (FV) infection in mice. Depletion of Treg or neutralization of IL-10 (but not TGF-β) resulted in increased NK cell anti-viral activity (109). **Figure 4A** illustrates various reported mechanisms of Treg modulation of NK cell cytolytic and pro-inflammatory functions.

Modulation of NK cells by Treg has also been reported to contribute to the maintenance of immune tolerance in models of allo-transplantation and autoimmune disease. For example, in mouse cardiac allografts, depletion of Treg resulted in increased graft infiltration by both NK cells and CD4^+^ T cells in association with more severe transplant vasculopathy. Of interest, the exacerbation of vasculopathy following Treg depletion was ameliorated by depletion of NK cells, implying an important role for Treg suppression on NK cells in allograft survival (110). A similar protective role for Treg-mediated NK cells suppression has been reported in bone marrow transplantation (111) and autoimmune hepatitis (112).

In contrast to the settings described above, the crosstalk between Treg and NK cells within the uterus during pregnancy appears to form a positive feedback loop in which depletion or dysfunction of either cell type can result in miscarriage (113). The canonical pro-inflammatory role of NK cells has also been challenged in autoimmune disease. While NK cells can be characterized as pathogenic through direct cytotoxicity and production of inflammatory cytokines, they may regulate adverse immune response by eliminating autoreactive immune cells and by serving as a source of anti-inflammatory mediators such as IL-10, TGF-β, and IL-4 (114). Thus, the combined immunological roles of NK cells and Treg are likely to be complex and highly contextual, necessitating more disease-specific research in human subjects to better understand the likely effects of Treg-based therapies on NK cell-mediated innate immune responses. This may be of particular interest in auto-inflammatory diseases in which sterile inflammation and dysfunctional innate immunity occur in the absence of autoantibodies and autoreactive T cells (115, 116).

### Natural killer T cells

Natural killer T (NKT) cells are a specialized type of innate-like lymphocytes, yet have the property of both NK cells and T cells by recognizing glycolipid ligands bound to the MHC class I-like protein CD1d via a restricted T cell receptor (TCR) repertoire and by widely participating in immune responses to infection, cancer, transplantation and fetal-maternal immunity (117). It was shown 20 years ago that human CD4^+^CD25^+^ Treg suppressed Vα24^+^NKT cell proliferation, cytotoxicity to tumor cells lines and cytokine production (IFN-γ, IL-4, IL-10, and IL-13) upon stimulation by α-galactosylceramide (αGalCer)-pulsed Mo-DCs. This suppression was dependent on direct contact between Treg and NKT cells and could be neutralized by antibody against ICAM but not IL-10 or TGF-β (118). Subsequently, Venken et al. reported that Treg suppressed proliferation and modulated cytokine production of human invariant (i)NKT cells in response to glycolipid or to innate stimuli. Conversely, Treg were induced to produce high amounts of IL-10 following contact with stimulated iNKT cells, indicating cross-talk between the two cell types (119). Evidence for direct Treg/NKT cell cross-talk *in vivo* has been reported by Hua et al., who demonstrated that CD1d deficient mice had reduced Treg number and function in the liver and that adoptively transferred Treg ameliorated NKT cell-mediated liver injury in a CD1d-dependent manner (120). In co-cultures of blood-derived human cells, induced Treg were shown to increase the proportion of CD4^+^ NKT cells with lower cytotoxicity and pro-inflammatory cytokine production than their CD4^-^ counterparts. Of interest, αGalCer-stimulated PBMC cultures from cancer patients contained higher proportions of both CD4^+^ NKT cells and effector Treg than those of patients with benign tumors (121).

In addition to the findings from studies such as these that Treg suppress NKT cell activation and function, predominantly through contact-dependent interactions, there is also experimental evidence that activated NKT cells delete or suppress the counter-regulatory functions of Treg in settings such as allergic asthma and pregnancy loss (122, 123). Of interest, NKT cells were also reported to promote Treg expression of negative costimulatory receptors PD-1 and IL-10 production through secreting IL-4 (124), and NKT cell-secreted IL-2 helped sustain Treg survival (125). Type 2 NKT cells, a subset with a more diverse TCR repertoire, have been documented to have immunomodulatory potential and to protect from autoimmune disease (126). **Figure 4B** summarizes aspects of Treg/NKT cell cross-talk described above. Overall, there is compelling evidence for direct, clinically-relevant interactions between Treg and NKT cells that merit further investigation – particularly in relation to diseases and treatment strategies in which NKT cells participate in dictating the nature of tissue inflammation.

### Gamma Delta T cells

γδ T cells represent a T cell subset which express TCRs consisting of γ and δ chains rather than the canonical TCR composed of α and β chains. They are typically categorized as innate immune cells because their activation via the TCR is MHC-independent and because they also express a range of surface NK receptors which modulate their responses to stress-associated antigens (127). The effector functions of γδ T cells include target cell cytolysis and production of inflammatory cytokines including IFNγ, TNF, IL-17A and IL-6. Accumulating evidence indicates that Treg exert regulatory effects on γδ T cells. For example, in a mouse model of ischemic stroke, the beneficial effect of intracerebral administration of IL-10 was shown, in part, to be mediated by induction of FoxP3^+^ Treg to suppress IL-17A production by γδ T cells (128). Similarly, IL-10-dependent Treg effects have been shown to reduce γδ T cell proliferation in a model of spontaneous colitis (129), and to inhibit human γδ T cell IFNγ secretion in response to the *Mycobacterium tuberculosis* antigen ESAT-6 (130). Direct Treg suppression of proliferation and production of IL-17A and IFNγ by γδ T cells was also observed in a mouse model of mucosal inflammation, although the mechanism was not elucidated (131). Along with IL-10, TGF-β has also been reported to participate in Treg suppression γδ T cell responses. In patients with hepatocellular carcinoma (HCC), compromised anti-tumor potency of γδ T cells was observed and Treg isolated from HCC tissue were suppressive of IFNγ production and tumor cell cytotoxicity by γδ T cells. Importantly, these effects were abrogated by anti-IL-10 or anti-TGF-β1 antibodies (132).

The role of specific Treg subpopulations in suppressing γδ T cells has been investigated in some disease settings. In a mouse model of myocardial infarction (MI), Blanco-Domínguez et al. used depletion and adoptive transfer of Treg to demonstrate that CD69^+^ Treg, through CD39 ectonucleotidase activity, protected against myocardial inflammation by suppressive effects on γδ T cells including induction of apoptosis, inhibition of IL-17A production and reduced migration to infarcted myocardium. In human subjects, higher proportions of circulating CD69^+^ Treg were shown to be associated with reduced risk of re-hospitalization for heart failure following acute MI (133). In a model of respiratory mucosal injury, Faustino et al. observed that depletion of GATA3^+^ Treg expressing the IL-33 receptor, ST2, resulted in increased IL-17-secreting γδ T cells and exacerbation of allergic lung inflammation. Mechanistically, the suppressive effect of ST2-expressing Treg on pulmonary γδ T cells was dependent on Treg exposure to allergen peptide and IL-33 resulting in Treg-secretion of Ebi3 (a component of IL-35). The inhibition of γδ T cells was associated with decreased eosinophil recruitment to the lungs (134). **Figure 4C** provides a summary of Treg markers, pathways and mediators shown to be relevant their modulatory interactions with γδ T cells.

As for other innate immune effectors, γδ T cells may also compete with and inhibit Treg functions. For example, IL-23 secretion by γδ T cells was shown to reduce Treg numbers and suppression of CD4^+^ αβT cells during mouse autoimmune encephalomyelitis (135). Similarly, secretion of IFNγ by γδ T cells may suppress the induction of Treg differentiation from CD4^+^ T cells as well as Treg expansion and suppression of effector T cells *in vitro* and *in vivo* (136–138). Importantly, in some settings, γδ T cells may also mediate regulatory effects on immune responses (139). Nonetheless, the weight of experimental evidence to date indicates that Treg can serve an important role in regulating the potent pro-inflammatory effects of γδ T cells in a range of diseases and that development of Treg-based therapies that replicate this function may be a promising translational goal.

### Innate lymphoid cells

Although NK cells have been characterized as innate lymphoid cells for several decades, additional subtypes of bone marrow-derived cell types with lymphoid morphology and non-antigen specific immunological functions have been described more recently and termed ILC1, 2 and 3. These cells are present in lymphoid organs and blood but occur at higher concentrations at epithelial barriers and other tissue sites and play important roles in regulating the responses to infection or injury and in maintaining tissue homeostasis. They serve as a potent source of cytokines and can be viewed as serving as “helper cells” during evolving immune/inflammatory responses. The analogy to helper cells is strengthened by the associations of ILC1, 2 and 3 with production of signature cytokines that match those of CD4^+^ Th1, Th2 and Th17 cells respectively (140, 141).

Investigation of the interactions between Treg and ILC is in its infancy and we focus here on recent studies related to ILC2 which represent the most numerous ILC and serve to control extracellular parasitic infection and widely participate in mucosal immunity, by secreting type 2 cytokines including IL-4, IL-5, IL-9, IL-13. Rigas et al. have shown that human TGF-β-induced Treg suppressed IL-5 and IL-13 secretion by ILC2 through contact-dependent (ICOS/ICOS-L) and soluble (TGF-β and IL-10) mediators to control airway inflammation in a humanized model of ILC-2-dependent asthma. Interestingly, this effect was absent in naïve Treg (142). Similarly, in a mouse model of allergic asthma, Khumalo et al. demonstrated that Treg expression of the IL-4 receptor alpha was necessary for Treg-mediated suppression of ILC2 proliferation and secretion of IL-5 and IL-13 (143). In co-culture experiments informed by a mouse model of asthma, Krishnamoorthy et al. observed dose-dependent Treg suppression of IL-13 secretion by ILC-2, which was enhanced by Treg priming with the fatty acid derivative maresin 1 (144). Lui et al. observed increased proportions of ILC2 and related type 2 cytokines in PBMC from patients with allergic rhinitis which was accompanied by reductions in IL-35-producing Treg. Subsequently, IL-35 and induced IL-35-producing Treg were shown to suppress ILC2 in activation cultures. A role for ICOS/ICOSL in Treg suppression of ILC2 was also shown in this study (145). In another recent study of PBMC from allergic rhinitis patients, Treg-secreted IL-10, induced by co-culture with induced pluripotent stem cell-derived mesenchymal stem cells (iPSC-MSC), was shown to suppress ILC-2-secretion of IL-13, IL-9, and IL-5 in an ICOS/ICOSL-dependent manner (146). The key mediators of Treg/ILC2 interactions that have been described to date are summarized in **Figure 4D**.

The impact of Treg on ILC2 has been investigated in non-allergic pathological contexts as well. For example, in a mouse model of chronic pancreatitis, Treg depletion resulted in increased proportion of ILC2 cells, but not ILC1, in spleen and pancreatic tissue and concomitantly aggravated pancreatic fibrosis (147). Finally, in contrast to other studies described here, Treg and ILC2 were shown by Gao et al. to play complementary roles in reducing the severity of atherosclerosis in a mouse model. In this study, Treg tended to promote rather than suppress ILC2 and ILC2-derived IL-13 in a cell-contact and cytokine-based (IL-10 and TGF-β) manner (148). Notably, ILC2, much like Treg cells, express CD25 and are consumers of IL-2. A consequence of this is that ILC2 may be inadvertently expanded by administration of low-dose IL-2 intended to enhance Treg numbers *in vivo (* 149). Whether there is natural antagonism between Treg and ILC2 during immune responses through competition for IL-2 remains to be directly investigated. Interestingly, it has been reported that IL-2 produced by ILC3 aids in supporting regulatory functions of Treg cells to maintain gut tissue homeostasis (150), while also serving as a cofactor to ILC2 survival/proliferation in lung inflammation (151). Thus, in common with other innate immune cell types reviewed here, the interactions described to date between ILC and Treg reflect a range of mechanisms and outcomes. Nonetheless, consistent striking observations of direct Treg-mediated suppression of maladaptive ILC2 activity in asthma and allergy speak to a distinct area of clinical potential.

## Relevance to human diseases and translational opportunities

In contrast to the growing number of clinical trials of Treg-based therapies in patients with autoimmune diseases and organ transplants, little clinical translation has occurred to date of the capacity of Treg to modulate innate immune responses. Nonetheless, as we have reviewed in the preceding sections, there is a wealth of basic and pre-clinical evidence that specific Treg functional characteristics could be exploited as novel treatment strategy for innate immune-mediated inflammatory diseases (152). We conclude this review with short appraisals of evidence in favor of the development of Treg-based therapies for three common inflammatory conditions in which innate immune responses play a key role: type 2 diabetics (T2DM), ischemia reperfusion injury (IRI) and atherosclerosis.

Firstly, in the case of T2DM and its major complications, some human studies have observed numerical defects in circulating Treg in association with the presence or progression of diabetic nephropathy, retinopathy (153) and diabetic foot ulcer (154). In addition, animal studies have demonstrated that adoptive transfer or *in vivo* expansion of Treg ameliorate the severity of T2DM and its macro- and micro-vascular complications. For example, in diabetic pigs, intravenously injected Treg were shown by Guo et al. to protect tissue-engineered blood vessels from intimal hyperplasia by inhibiting inflammation and cell apoptosis and promoting endothelial progenitor cell mobilization (155). Other studies in rodent models of diabetes have demonstrated that adoptively transferred Treg migrate to retina and reduce neovascular retinopathy (156) or suppress obesity-linked insulin resistance and preserve kidney function (157). In a model of stroke in T2DM, endogenous expansion of Treg by a CD28 superagonist was found to increase M2-type macrophages and to alleviate ischemic brain injury (28). Similarly, *in vivo* expansion of Treg in mice with T2DM by injection of a novel hybrid cytokine bearing IL-2 and IL-33 activities, reduced diabetic kidney damage (158). Importantly, functional as well as numerical Treg deficits may also occur in T2DM. For example, Zhu et al. observed increased proportions of IL-17-producing Foxp3^+^Treg in T2DM patients compared to healthy patients which associated with higher body mass index (BMI) and the HbA1c levels (159). Similarly, Sheikh et al. reported that CD4^+^CD25^+^CD45RA^+^ Treg have comparable frequency but reduced suppressive potency in T2DM compared to non-diabetic controls (160). Overall, these and other studies provide evidence that augmentation of the autologous Treg repertoire in T2DM, either through ex vivo or endogenous expansion, has potential to prevent or reverse multiple severe diabetic complications.

Ischemia reperfusion injury refers to an acute compromise to the blood supply of an organ or tissue resulting in cellular damage or necrosis followed by a restoration of blood flow which further aggravates tissue injury by activating inflammatory processes. A significant role for Treg in alleviating inflammation has been demonstrated through Treg depletion and adoptive transfer in animal models of intestinal and renal IRI and of ischemic stroke (161, 162). In keeping with modulatory effects on mononuclear phagocytes summarized in an earlier section, ex vivo expanded Treg have also been shown to modulate the production of TNF and IL-1β by Kuffer cells and, thereby, protect against acute liver injury due to IRI (163). More widely studied has been the impact of Treg on myocardial IRI. A recent detailed review of this topic highlights that Treg, migrating to myocardial tissue after infarction through CCR5, the CXCR4-CXCL12 axis, and the Hippo pathway, may promote myocardial tissue repair through multiple mechanisms including modulation of macrophage phenotypes, direct interactions with cardiomyocytes and increasing collagen content and maturation in the infarct zone (164). A distinct heart-resident Helios^high^Nrp-1^high^ Treg population, which expresses Sparc and is sustained and expanded by IL-33/ST-2 interaction was also identified and proved to be the primary mediator of Treg-mediated repair following myocardial infarction (165). In acute kidney injury, a CXCR3^+^ Treg subpopulation was found to be recruited to kidney tissue following reperfusion and to correlate with lower renal tissue injury and better kidney function (166). As evidenced by this predominantly pre-clinical literature, therapeutic strategies aimed at expanding or delivering pro-repair Treg to the site of IRI-related tissue injury have promise for limiting the consequences to brain (162), heart, kidney, liver, intestine and other organs in clinical settings. However, successful translational strategies in this area will likely require highly stable Treg-based therapies with distinct migratory and effector functions.

Atherosclerosis refers to the formation of cholesterol-containing plaques in arterial blood vessels, which may result in severe acute or chronic cardiovascular conditions due to reduced blood flow to tissue or rupture leading to thrombosis. Following initial changes to endothelial cells, increasing evidence indicates that progression of atherosclerotic plaques is driven by inflammatory responses. Indeed, three recent clinical trials of anti-inflammatory agents have proven beneficial effects in patients with atherosclerosis, including reductions in cardiovascular events and mortality (167–169). A number of studies have provided evidence for anti-inflammatory and athero-protective roles for Treg in atherosclerosis, including correlations between disease progression and defects of Treg numbers (170) or function (171). In animal models, Sharma et al, provided more direct evidence that Treg protect from rupture and promote regression of atherosclerotic plaques through modulatory effects on macrophages (47). Adoptively transferred Treg have also been found to contribute to reduced size and contraction of atherosclerotic plaques in hypercholesterolemic mice (172, 173). Studies such as these provide convincing evidence that Treg-enhancing approaches could be of value as a therapeutic strategy for reversing atherosclerosis. It is worth noting, however, that challenges will arise in maintaining the stability and adaptability of Treg in the pro-inflammatory setting of atherosclerosis (174, 175). In this regard, Treg within atherosclerotic aortas have been reported to have a high expression of Foxp3 but also low CD25 levels, expression of Th1-associated factors IFN-γ and T-bet and compromised suppressive potency (176). Understanding Treg metabolism under hypoxic conditions may help to address this issue.

## Conclusion

As we have described here, the capacity for Treg to suppress innate immune responses and to ameliorate acute and chronic diseases involving non antigen-specific inflammation is well established. Furthermore, many mechanistic details of the specific modulatory effects of Treg and Treg subpopulations on individual innate immune effectors have been identified in experimental animals as well as human cells. That this body of knowledge could be exploited to develop Treg-enhancing strategies for a much broader range of conditions than that which they have, to date, been applied seems self-evident. Importantly, Treg cell therapies have proven to be safe in early phase clinical trials and are now in the process of overcoming several important obstacles to their more effective use in autoimmune diseases and organ transplants. While this progress should provide a valuable platform for extending Treg therapies to diseases driven primarily by innate immune responses, additional challenges related to tissue targeting, *in vivo* stability and selection for the required effector mechanisms will almost certainly require an extensive amount of focused research and innovation. Nonetheless, we propose that the existing evidence is sufficient to support a greater investment of time and resources in the development of interventions to augment Treg modulation of innate immunity.

## Author contributions

QO: Conceptualization, Funding acquisition, Writing – original draft, Writing – review and editing. RP: Funding acquisition, Writing – original draft. MG: Conceptualization, Funding acquisition, Supervision, Writing – review and editing.

## References

[1] Sakaguchi S Mikami N Wing JB Tanaka A Ichiyama K Ohkura N. Regulatory T cells and human disease. Annu Rev Immunol (2020) 38:541–66. doi: 10.1146/annurev-immunol-042718-041717 32017635

[2] Okeke EB Uzonna JE. The pivotal role of regulatory T cells in the regulation of innate immune cells. Front Immunol (2019) 10:680. doi: 10.3389/fimmu.2019.00680 31024539 PMC6465517

[3] McInnes IB Gravallese EM. Immune-mediated inflammatory disease therapeutics: past, present and future. Nat Rev Immunol (2021) 21(10):680–6. doi: 10.1038/s41577-021-00603-1 PMC8436867 34518662

[4] Romano M Fanelli G Albany CJ Giganti G Lombardi G. Past, present, and future of regulatory T cell therapy in transplantation and autoimmunity. Front Immunol (2019) 10:43. doi: 10.3389/fimmu.2019.00043 30804926 PMC6371029

[5] Liu Y Carlsson R Comabella M Wang J Kosicki M Carrion B. FoxA1 directs the lineage and immunosuppressive properties of a novel regulatory T cell population in EAE and MS. Nat Med (2014) 20(3):272–82. doi: 10.1038/nm.3485 24531377

[6] Giganti G Atif M Mohseni Y Mastronicola D Grageda N Povoleri GA. Treg cell therapy: How cell heterogeneity can make the difference. Eur J Immunol (2021) 51(1):39–55. doi: 10.1002/eji.201948131 33275279

[7] Negi N Griffin MD. Effects of mesenchymal stromal cells on regulatory T cells: Current understanding and clinical relevance. Stem Cells (2020) 38(5):596–605. doi: 10.1002/stem.3151 31995249 PMC7217190

[8] Grosse L Hoogenboezem T Ambrée O Bellingrath S Jörgens S de Wit HJ. Deficiencies of the T and natural killer cell system in major depressive disorder: T regulatory cell defects are associated with inflammatory monocyte activation. Brain Behav Immun (2016) 54:38–44. doi: 10.1016/j.bbi.2015.12.003 26674997

[9] Liu L Zhang Q Chen P Guo N Song A Huang X. Foxp3Helios regulatory T cells are associated with monocyte subsets and their PD-1 expression during acute HIV-1 infection. BMC Immunol (2019) 20(1):38. doi: 10.1186/s12865-019-0319-7 31651258 PMC6813100

[10] Skuljec J Cabanski M Surdziel E Lachmann N Brennig S Pul R. Monocyte/macrophage lineage commitment and distribution are affected by the lack of regulatory T cells in scurfy mice. Eur J Immunol (2016) 46(7):1656–68. doi: 10.1002/eji.201546200 27130185

[11] Hand LE Gray KJ Dickson SH Simpkins DA Ray DW Konkel JE. Regulatory T cells confer a circadian signature on inflammatory arthritis. Nat Commun (2020) 11(1):1658. doi: 10.1038/s41467-020-15525-0 32245954 PMC7125185

[12] Fu Y Yi S Wu J Jimenez E Simond D Hawthorne WJ. *In vitro* suppression of xenoimmune-mediated macrophage activation by human CD4+CD25+ regulatory T cells. Transplantation (2008) 86(6):865–74. doi: 10.1097/TP.0b013e31818530fd 18813112

[13] Robinson TO Zhang M Ochsenbauer C Smythies LE Cron RQ. CD4 regulatory T cells augment HIV-1 expression of polarized M1 and M2 monocyte derived macrophages. Virology (2017) 504:79–87. doi: 10.1016/j.virol.2017.01.018 28157548 PMC5687253

[14] Xie L Choudhury GR Winters A Yang S-H Jin K. Cerebral regulatory T cells restrain microglia/macrophage-mediated inflammatory responses via IL-10. Eur J Immunol (2015) 45(1):180–91. doi: 10.1002/eji.201444823 PMC4293323 25329858

[15] Proto JD Doran AC Gusarova G Yurdagul A Sozen E Subramanian M. Regulatory T cells promote macrophage efferocytosis during inflammation resolution. Immunity (2018) 49(4):666–77. doi: 10.1016/j.immuni.2018.07.015 PMC6192849 30291029

[16] Tiemessen MM Jagger AL Evans HG van Herwijnen MJC John S Taams LS. CD4+CD25+Foxp3+ regulatory T cells induce alternative activation of human monocytes/macrophages. Proc Natl Acad Sci U S A. (2007) 104(49):19446–51. doi: 10.1073/pnas.0706832104 PMC2148309 18042719

[17] Liu G Ma H Qiu L Li L Cao Y Ma J. Phenotypic and functional switch of macrophages induced by regulatory CD4+CD25+ T cells in mice. Immunol Cell Biol (2011) 89(1):130–42. doi: 10.1038/icb.2010.70 20514074

[18] Hou X-X Wang X-Q Zhou W-J Li D-J. Regulatory T cells induce polarization of pro-repair macrophages by secreting sFGL2 into the endometriotic milieu. Commun Biol (2021) 4(1):499. doi: 10.1038/s42003-021-02018-z 33893391 PMC8065041

[19] Wara AK Rawal S Yang X Pérez-Cremades D Sachan M Chen J. KLF10 deficiency in CD4 T cells promotes atherosclerosis progression by altering macrophage dynamics. Atherosclerosis (2022) 359:27–41. doi: 10.1016/j.atherosclerosis.2022.08.019 36174463

[20] Yang K Zhang Y Xu C Li X Li D. mTORC1 signaling is crucial for regulatory T cells to suppress macrophage-mediated inflammatory response after acute myocardial infarction. Immunol Cell Biol (2016) 94(3):274–84. doi: 10.1038/icb.2015.88 26437770

[21] Hu H Wu J Cao C Ma L. Exosomes derived from regulatory T cells ameliorate acute myocardial infarction by promoting macrophage M2 polarization. IUBMB Life (2020) 72(11):2409–19. doi: 10.1002/iub.2364 32842172

[22] Zhou J Li X Wu X Zhang T Zhu Q Wang X. Exosomes released from tumor-associated macrophages transfer miRNAs that induce a treg/th17 cell imbalance in epithelial ovarian cancer. Cancer Immunol Res (2018) 6(12):1578–92. doi: 10.1158/2326-6066.CIR-17-0479 30396909

[23] Sun W Wei F-Q Li W-J Wei J-W Zhong H Wen Y-H. A positive-feedback loop between tumour infiltrating activated Treg cells and type 2-skewed macrophages is essential for progression of laryngeal squamous cell carcinoma. Br J Cancer (2017) 117(11):1631–43. doi: 10.1038/bjc.2017.329 PMC5729431 28949956

[24] Li J Kim SY Lainez NM Coss D Nair MG. Macrophage-regulatory T cell interactions promote type 2 immune homeostasis through resistin-like molecule α. Front Immunol (2021) 12:710406. doi: 10.3389/fimmu.2021.710406 34349768 PMC8327085

[25] Guo Z Wen Z Qin A Zhou Y Liao Z Liu Z. Antisense oligonucleotide treatment enhances the recovery of acute lung injury through IL-10-secreting M2-like macrophage-induced expansion of CD4+ regulatory T cells. J Immunol (2013) 190(8):4337–48. doi: 10.4049/jimmunol.1203233 PMC3619531 23514739

[26] Lu J Lv S Pang J Qin T Yang Y Lu W. M2c macrophages protect mice from adriamycin-induced nephropathy by upregulating CD62L in tregs. Mediators Inflamm (2022) 2022:1153300. doi: 10.1155/2022/1153300 36262548 PMC9576407

[27] Romano M Fanelli G Tan N Nova-Lamperti E McGregor R Lechler RI. Expanded regulatory T cells induce alternatively activated monocytes with a reduced capacity to expand T helper-17 cells. Front Immunol (2018) 9:1625. doi: 10.3389/fimmu.2018.01625 30079063 PMC6062605

[28] Cai Y Xu T-T Lu C-Q Ma Y-Y Chang D Zhang Y. Endogenous regulatory T cells promote M2 macrophage phenotype in diabetic stroke as visualized by optical imaging. Transl Stroke Res (2021) 12(1):136–46. doi: 10.1007/s12975-020-00808-x 32240524

[29] Buechler MB Fu W Turley SJ. Fibroblast-macrophage reciprocal interactions in health, fibrosis, and cancer. Immunity (2021) 54(5):903–15. doi: 10.1016/j.immuni.2021.04.021 33979587

[30] Misharin AV Morales-Nebreda L Reyfman PA Cuda CM Walter JM McQuattie-Pimentel AC. Monocyte-derived alveolar macrophages drive lung fibrosis and persist in the lung over the life span. J Exp Med (2017) 214(8):2387–404. doi: 10.1084/jem.20162152 PMC5551573 28694385

[31] Tan-Garcia A Lai F Sheng Yeong JP Irac SE Ng PY Msallam R. Liver fibrosis and CD206 macrophage accumulation are suppressed by anti-GM-CSF therapy. JHEP Rep (2020) 2(1):100062. doi: 10.1016/j.jhepr.2019.11.006 32039403 PMC7005658

[32] Kim M-G Kim SC Ko YS Lee HY Jo S-K Cho W. The role of M2 macrophages in the progression of chronic kidney disease following acute kidney injury. PloS One (2015) 10(12):e0143961. doi: 10.1371/journal.pone.0143961 26630505 PMC4667939

[33] Bhandari R Ball MS Martyanov V Popovich D Schaafsma E Han S. Profibrotic activation of human macrophages in systemic sclerosis. Arthritis Rheumatol (2020) 72(7):1160–9. doi: 10.1002/art.41243 PMC7329566 32134204

[34] Song J Gong Y-H Yan X Liu Y Zhang M Luo J. Regulatory T cells accelerate the repair process of renal fibrosis by regulating mononuclear macrophages. Am J Med Sci (2021) 361(6):776–85. doi: 10.1016/j.amjms.2021.01.022 33667434

[35] Cucak H Nielsen Fink L Højgaard Pedersen M Rosendahl A. Enalapril treatment increases T cell number and promotes polarization towards M1-like macrophages locally in diabetic nephropathy. Int Immunopharmacol (2015) 25(1):30–42. doi: 10.1016/j.intimp.2015.01.003 25598292

[36] Sun H Tian J Xian W Xie T Yang X. Pentraxin-3 attenuates renal damage in diabetic nephropathy by promoting M2 macrophage differentiation. Inflammation (2015) 38(5):1739–47. doi: 10.1007/s10753-015-0151-z 25761429

[37] Zhang M Zhang S. T cells in fibrosis and fibrotic diseases. Front Immunol (2020) 11:1142. doi: 10.3389/fimmu.2020.01142 32676074 PMC7333347

[38] Bansal SS Ismahil MA Goel M Zhou G Rokosh G Hamid T. Dysfunctional and proinflammatory regulatory T-lymphocytes are essential for adverse cardiac remodeling in ischemic cardiomyopathy. Circulation (2019) 139(2):206–21. doi: 10.1161/CIRCULATIONAHA.118.036065 PMC6322956 30586716

[39] Estes JD Wietgrefe S Schacker T Southern P Beilman G Reilly C. Simian immunodeficiency virus-induced lymphatic tissue fibrosis is mediated by transforming growth factor beta 1-positive regulatory T cells and begins in early infection. J Infect Dis (2007) 195(4):551–61. doi: 10.1086/510852 17230415

[40] Seyran M Melanie S Philip S Amiq G Fabian B. Allies or enemies? The effect of regulatory T cells and related T lymphocytes on the profibrotic environment in bleomycin-injured lung mouse models. Clin Exp Med (2022) 23(4):1075–88. doi: 10.1007/s10238-022-00945-7 PMC10390389 36403186

[41] Leite Dantas R Masemann D Schied T Bergmeier V Vogl T Loser K. Macrophage-mediated psoriasis can be suppressed by regulatory T lymphocytes. J Pathol (2016) 240(3):366–77. doi: 10.1002/path.4786 27555499

[42] Kamata M Tada Y. Dendritic cells and macrophages in the pathogenesis of psoriasis. Front Immunol (2022) 13:941071. doi: 10.3389/fimmu.2022.941071 35837394 PMC9274091

[43] Bolivar-Wagers S Larson JH Jin S Blazar BR. Cytolytic CD4 and CD8 regulatory T-cells and implications for developing immunotherapies to combat graft-versus-host disease. Front Immunol (2022) 13:864748. doi: 10.3389/fimmu.2022.864748 35493508 PMC9040077

[44] Venet F Pachot A Debard A-L Bohe J Bienvenu J Lepape A. Human CD4+CD25+ regulatory T lymphocytes inhibit lipopolysaccharide-induced monocyte survival through a Fas/Fas ligand-dependent mechanism. J Immunol (2006) 177(9):6540–7. doi: 10.4049/jimmunol.177.9.6540 17056586

[45] Jagger AL Evans HG Walter GJ Gullick NJ Menon B Ballantine LE. FAS/FAS-L dependent killing of activated human monocytes and macrophages by CD4+CD25- responder T cells, but not CD4+CD25+ regulatory T cells. J Autoimmun (2012) 38(1):29–38. doi: 10.1016/j.jaut.2011.11.015 22197557

[46] Grossman WJ Verbsky JW Barchet W Colonna M Atkinson JP Ley TJ. Human T regulatory cells can use the perforin pathway to cause autologous target cell death. Immunity (2004) 21(4):589–601. doi: 10.1016/j.immuni.2004.09.002 15485635

[47] Sharma M Schlegel MP Afonso MS Brown EJ Rahman K Weinstock A. Regulatory T cells license macrophage pro-resolving functions during atherosclerosis regression. Circ Res (2020) 127(3):335–53. doi: 10.1161/CIRCRESAHA.119.316461 PMC7367765 32336197

[48] Lewkowicz N Mycko MP Przygodzka P Ćwiklińska H Cichalewska M Matysiak M. Induction of human IL-10-producing neutrophils by LPS-stimulated Treg cells and IL-10. Mucosal Immunol (2016) 9(2):364–78. doi: 10.1038/mi.2015.66 26220165

[49] Lewkowicz N Klink M Mycko MP Lewkowicz P. Neutrophil–CD4+CD25+ T regulatory cell interactions: a possible new mechanism of infectious tolerance. Immunobiology (2013) 218(4):455–64. doi: 10.1016/j.imbio.2012.05.029 22749980

[50] Okeke EB Mou Z Onyilagha N Jia P Gounni AS Uzonna JE. Deficiency of phosphatidylinositol 3-kinase δ Signaling leads to diminished numbers of regulatory T cells and increased neutrophil activity resulting in mortality due to endotoxic shock. J Immunol (2017) 199(3):1086–95. doi: 10.4049/jimmunol.1600954 28659355

[51] Hu J Kang H Liu C Hu P Yang M Zhou F. Regulatory T cells could improve intestinal barrier dysfunction in heatstroke. Inflammation (2019) 42(4):1228–38. doi: 10.1007/s10753-019-00983-6 30820807

[52] Umeshappa CS Solé P Surewaard BGJ Yamanouchi J Mohapatra S Uddin MM. Liver-specific T regulatory type-1 cells program local neutrophils to suppress hepatic autoimmunity via CRAMP. Cell Rep (2021) 34(13):108919. doi: 10.1016/j.celrep.2021.108919 33789099

[53] Himmel ME Crome SQ Ivison S Piccirillo C Steiner TS Levings MK. Human CD4+ FOXP3+ regulatory T cells produce CXCL8 and recruit neutrophils. Eur J Immunol (2011) 41(2):306–12. doi: 10.1002/eji.201040459 21268001

[54] Qi X Yu Y Sun R Huang J Liu L Yang Y. Identification and characterization of neutrophil heterogeneity in sepsis. Crit Care (2021) 25(1):50. doi: 10.1186/s13054-021-03481-0 33549126 PMC7865119

[55] Tao M He Y Li L Li Y Liao W Nie H. Identification and validation of immune-associated NETosis subtypes and biomarkers in anti-neutrophil cytoplasmic antibody associated glomerulonephritis. Front Immunol (2023) 14:1177968. doi: 10.3389/fimmu.2023.1177968 37465687 PMC10351423

[56] Bieber K Sun S Witte M Kasprick A Beltsiou F Behnen M. Regulatory T cells suppress inflammation and blistering in pemphigoid diseases. Front Immunol (2017) 8:1628. doi: 10.3389/fimmu.2017.01628 29225603 PMC5705561

[57] Wang H Zhang H Wang Y Brown ZJ Xia Y Huang Z. Regulatory T-cell and neutrophil extracellular trap interaction contributes to carcinogenesis in non-alcoholic steatohepatitis. J Hepatol (2021) 75(6):1271–83. doi: 10.1016/j.jhep.2021.07.032 PMC12888775 34363921

[58] Wilson AS Randall KL Pettitt JA Ellyard JI Blumenthal A Enders A. Neutrophil extracellular traps and their histones promote Th17 cell differentiation directly via TLR2. Nat Commun (2022) 13(1):528. doi: 10.1038/s41467-022-28172-4 35082281 PMC8792063

[59] Moreau JM Dhariwala MO Gouirand V Boda DP Boothby IC Lowe MM. Regulatory T cells promote innate inflammation after skin barrier breach via TGF-β activation. Sci Immunol (2021) 6(62):eabg2329. doi: 10.1126/sciimmunol.abg2329 34452925 PMC8958044

[60] Sibilano R Frossi B Suzuki R D'Incà F Gri G Piconese S. Modulation of FcϵRI-dependent mast cell response by OX40L via Fyn, PI3K, and RhoA. J Allergy Clin Immunol (2012) 130(3):751–760.e2. doi: 10.1016/j.jaci.2012.03.032 22564682

[61] Ganeshan K Johnston LK Bryce PJ. TGF-β1 limits the onset of innate lung inflammation by promoting mast cell-derived IL-6. J Immunol (2013) 190(11):5731–8. doi: 10.4049/jimmunol.1203362 PMC3725733 23630359

[62] Ganeshan K Bryce PJ. Regulatory T cells enhance mast cell production of IL-6 via surface-bound TGF-β. J Immunol (2012) 188(2):594–603. doi: 10.4049/jimmunol.1102389 22156492 PMC3253181

[63] Macey MR Sturgill JL Morales JK Falanga YT Morales J Norton SK. IL-4 and TGF-beta 1 counterbalance one another while regulating mast cell homeostasis. J Immunol (2010) 184(9):4688–95. doi: 10.4049/jimmunol.0903477 PMC3339193 20304823

[64] Lu L-F Lind EF Gondek DC Bennett KA Gleeson MW Pino-Lagos K. Mast cells are essential intermediaries in regulatory T-cell tolerance. Nature (2006) 442(7106):997–1002. doi: 10.1038/nature05010 16921386

[65] Eller K Wolf D Huber JM Metz M Mayer G McKenzie ANJ. IL-9 production by regulatory T cells recruits mast cells that are essential for regulatory T cell-induced immune suppression. J Immunol (2011) 186(1):83–91. doi: 10.4049/jimmunol.1001183 21115728 PMC3227733

[66] Blankenhaus B Reitz M Brenz Y Eschbach M-L Hartmann W Haben I. Foxp3^+^ regulatory T cells delay expulsion of intestinal nematodes by suppression of IL-9-driven mast cell activation in BALB/c but not in C57BL/6 mice. PloS Pathog (2014) 10(2):e1003913. doi: 10.1371/journal.ppat.1003913 24516385 PMC3916398

[67] Hershko AY Suzuki R Charles N Alvarez-Errico D Sargent JL Laurence A. Mast cell interleukin-2 production contributes to suppression of chronic allergic dermatitis. Immunity (2011) 35(4):562–71. doi: 10.1016/j.immuni.2011.07.013 PMC3432919 21982597

[68] Zhang W Wu K He W Gao Y Huang W Lin X. Transforming growth factor beta 1 plays an important role in inducing CD4(+)CD25(+)forhead box P3(+) regulatory T cells by mast cells. Clin Exp Immunol (2010) 161(3):490–6. doi: 10.1111/j.1365-2249.2010.04190.x PMC2962966 20550544

[69] Forward NA Furlong SJ Yang Y Lin T-J Hoskin DW. Mast cells down-regulate CD4+CD25+ T regulatory cell suppressor function via histamine H1 receptor interaction. J Immunol (2009) 183(5):3014–22. doi: 10.4049/jimmunol.0802509 19667094

[70] Benede S Tordesillas L Berin C. Demonstration of distinct pathways of mast cell-dependent inhibition of Treg generation using murine bone marrow-derived mast cells. Allergy (2020) 75(8):2088–91. doi: 10.1111/all.14267 32147829

[71] Piconese S Gri G Tripodo C Musio S Gorzanelli A Frossi B. Mast cells counteract regulatory T-cell suppression through interleukin-6 and OX40/OX40L axis toward Th17-cell differentiation. Blood (2009) 114(13):2639–48. doi: 10.1182/blood-2009-05-220004 19643985

[72] Krajewska NM Fiancette R Oo YH. Interplay between mast cells and regulatory T cells in immune-mediated cholangiopathies. Int J Mol Sci (2022) 23(11):5872. doi: 10.3390/ijms23115872 35682552 PMC9180565

[73] Bellini R Bonacina F Norata GD. Crosstalk between dendritic cells and T lymphocytes during atherogenesis: Focus on antigen presentation and break of tolerance. Front Cardiovasc Med (2022) 9:934314. doi: 10.3389/fcvm.2022.934314 35966516 PMC9365967

[74] Klaus T Wilson AS Vicari E Hadaschik E Klein M Helbich SS. Impaired regulatory T cell-dendritic cell interactions contribute to autoimmunity in leukocyte adhesion deficiency type-1. JCI Insight (2022) 7(24):e162580. doi: 10.1172/jci.insight.162580 36346673 PMC9869970

[75] Raker VK Domogalla MP Steinbrink K. Tolerogenic dendritic cells for regulatory T cell induction in man. Front Immunol (2015) 6:569. doi: 10.3389/fimmu.2015.00569 26617604 PMC4638142

[76] Cederbom L Hall H Ivars F. CD4+CD25+ regulatory T cells down-regulate co-stimulatory molecules on antigen-presenting cells. Eur J Immunol (2000) 30(6):1538–43. doi: 10.1002/1521-4141(200006)30:6<1538::AID-IMMU1538>3.0.CO;2-X 10898488

[77] Qureshi OS Zheng Y Nakamura K Attridge K Manzotti C Schmidt EM. Trans-endocytosis of CD80 and CD86: a molecular basis for the cell-extrinsic function of CTLA-4. Science (2011) 332(6029):600–3. doi: 10.1126/science.1202947 PMC3198051 21474713

[78] Gu P Gao JF D'Souza CA Kowalczyk A Chou K-Y Zhang L. Trogocytosis of CD80 and CD86 by induced regulatory T cells. Cell Mol Immunol (2012) 9(2):136–46. doi: 10.1038/cmi.2011.62 PMC4002813 22307040

[79] Tekguc M Wing JB Osaki M Long J Sakaguchi S. Treg-expressed CTLA-4 depletes CD80/CD86 by trogocytosis, releasing free PD-L1 on antigen-presenting cells. Proc Natl Acad Sci U S A. (2021) 118(30):e2023739118. doi: 10.1073/pnas.2023739118 34301886 PMC8325248

[80] Dhainaut M Coquerelle C Uzureau S Denoeud J Acolty V Oldenhove G. Thymus-derived regulatory T cells restrain pro-inflammatory Th1 responses by downregulating CD70 on dendritic cells. EMBO J (2015) 34(10):1336–48. doi: 10.15252/embj.201490312 PMC4491995 25787857

[81] Dai H Pena A Bauer L Williams A Watkins SC Camirand G. Treg suppression of immunity within inflamed allogeneic grafts. JCI Insight (2022) 7(16):e160579. doi: 10.1172/jci.insight.160579 35881490 PMC9462475

[82] Zhou P Xu J Dai M Shi Y Wu G Fang Y. The immunosuppressive effects of CD4 CD25 regulatory T cells on dendritic cells in patients with chronic hepatitis B. J Viral Hepat (2018) 25(6):733–41. doi: 10.1111/jvh.12863 29345851

[83] Larmonier N Marron M Zeng Y Cantrell J Romanoski A Sepassi M. Tumor-derived CD4(+)CD25(+) regulatory T cell suppression of dendritic cell function involves TGF-beta and IL-10. Cancer Immunol Immunother (2007) 56(1):48–59. doi: 10.1007/s00262-006-0160-8 16612596 PMC11030031

[84] Haller S Duval A Migliorini R Stevanin M Mack V Acha-Orbea H. Interleukin-35-producing CD8α Dendritic cells acquire a tolerogenic state and regulate T cell function. Front Immunol (2017) 8:98. doi: 10.3389/fimmu.2017.00098 28228759 PMC5296329

[85] Zhang X Zheng P Prestwood TR Zhang H Carmi Y Tolentino LL. Human regulatory dendritic cells develop from monocytes in response to signals from regulatory and helper T cells. Front Immunol (2020) 11:1982. doi: 10.3389/fimmu.2020.01982 32973804 PMC7461788

[86] Tung SL Boardman DA Sen M Letizia M Peng Q Cianci N. Regulatory T cell-derived extracellular vesicles modify dendritic cell function. Sci Rep (2018) 8(1):6065. doi: 10.1038/s41598-018-24531-8 29666503 PMC5904112

[87] Tang Q Henriksen KJ Bi M Finger EB Szot G Ye J. *In vitro*-expanded antigen-specific regulatory T cells suppress autoimmune diabetes. J Exp Med (2004) 199(11):1455–65. doi: 10.1084/jem.20040139 PMC2211775 15184499

[88] Tarbell KV Yamazaki S Olson K Toy P Steinman RM. CD25+ CD4+ T cells, expanded with dendritic cells presenting a single autoantigenic peptide, suppress autoimmune diabetes. J Exp Med (2004) 199(11):1467–77. doi: 10.1084/jem.20040180 PMC2211787 15184500

[89] Selck C Dominguez-Villar M. Antigen-specific regulatory T cell therapy in autoimmune diseases and transplantation. Front Immunol (2021) 12:661875. doi: 10.3389/fimmu.2021.661875 34054826 PMC8160309

[90] Akkaya B Oya Y Akkaya M Al Souz J Holstein AH Kamenyeva O. Regulatory T cells mediate specific suppression by depleting peptide-MHC class II from dendritic cells. Nat Immunol (2019) 20(2):218–31. doi: 10.1038/s41590-018-0280-2 PMC6402611 30643268

[91] Liang B Workman C Lee J Chew C Dale BM Colonna L. Regulatory T cells inhibit dendritic cells by lymphocyte activation gene-3 engagement of MHC class II. J Immunol (2008) 180(9):5916–26. doi: 10.4049/jimmunol.180.9.5916 18424711

[92] Chen J Ganguly A Mucsi AD Meng J Yan J Detampel P. Strong adhesion by regulatory T cells induces dendritic cell cytoskeletal polarization and contact-dependent lethargy. J Exp Med (2017) 214(2):327–38. doi: 10.1084/jem.20160620 PMC5294852 28082358

[93] Yan J Liu B Shi Y Qi H. Class II MHC-independent suppressive adhesion of dendritic cells by regulatory T cells in vivo. J Exp Med (2017) 214(2):319–26. doi: 10.1084/jem.20160629 PMC5294853 28082359

[94] Comrie WA Li S Boyle S Burkhardt JK. The dendritic cell cytoskeleton promotes T cell adhesion and activation by constraining ICAM-1 mobility. J Cell Biol (2015) 208(4):457–73. doi: 10.1083/jcb.201406120 PMC4332244 25666808

[95] Mittelbrunn M Molina A Escribese MM Yáñez-Mó M Escudero E Ursa A. VLA-4 integrin concentrates at the peripheral supramolecular activation complex of the immune synapse and drives T helper 1 responses. Proc Natl Acad Sci U S A. (2004) 101(30):11058–63. doi: 10.1073/pnas.0307927101 PMC503740 15263094

[96] Sarris M Andersen KG Randow F Mayr L Betz AG. Neuropilin-1 expression on regulatory T cells enhances their interactions with dendritic cells during antigen recognition. Immunity (2008) 28(3):402–13. doi: 10.1016/j.immuni.2008.01.012 PMC2726439 18328743

[97] Li R Li H Yang X Hu H Liu P Liu H. Crosstalk between dendritic cells and regulatory T cells: Protective effect and therapeutic potential in multiple sclerosis. Front Immunol (2022) 13:970508. doi: 10.3389/fimmu.2022.970508 36177043 PMC9513370

[98] Sayitoglu EC Freeborn RA Roncarolo MG. The yin and yang of type 1 regulatory T cells: from discovery to clinical application. Front Immunol (2021) 12:693105. doi: 10.3389/fimmu.2021.693105 34177953 PMC8222711

[99] Mfarrej B Tresoldi E Stabilini A Paganelli A Caldara R Secchi A. Generation of donor-specific Tr1 cells to be used after kidney transplantation and definition of the timing of their in *vivo* infusion in the presence of immunosuppression. J Transl Med (2017) 15(1):40. doi: 10.1186/s12967-017-1133-8 28222739 PMC5319067

[100] Gregori S Tomasoni D Pacciani V Scirpoli M Battaglia M Magnani CF. Differentiation of type 1 T regulatory cells (Tr1) by tolerogenic DC-10 requires the IL-10-dependent ILT4/HLA-G pathway. Blood (2010) 116(6):935–44. doi: 10.1182/blood-2009-07-234872 20448110

[101] Comi M Avancini D Santoni de Sio F Villa M Uyeda MJ Floris M. Coexpression of CD163 and CD141 identifies human circulating IL-10-producing dendritic cells (DC-10). Cell Mol Immunol (2020) 17(1):95–107. doi: 10.1038/s41423-019-0218-0 PMC6952411 30842629

[102] Zeng H Zhang R Jin B Chen L. Type 1 regulatory T cells: a new mechanism of peripheral immune tolerance. Cell Mol Immunol (2015) 12(5):566–71. doi: 10.1038/cmi.2015.44 PMC4579656 26051475

[103] Chang W-C Li C-H Chu L-H Huang P-S Sheu B-C Huang S-C. Regulatory T cells suppress natural killer cell immunity in patients with human cervical carcinoma. Int J Gynecol Cancer (2016) 26(1):156–62. doi: 10.1097/IGC.0000000000000578 26512789

[104] Pedroza-Pacheco I Madrigal A Saudemont A. Interaction between natural killer cells and regulatory T cells: perspectives for immunotherapy. Cell Mol Immunol (2013) 10(3):222–9. doi: 10.1038/cmi.2013.2 PMC4012769 23524654

[105] Chen Y Sun R Wu X Cheng M Wei H Tian Z. CD4+CD25+ Regulatory T cells inhibit natural killer cell hepatocytotoxicity of hepatitis B virus transgenic mice via membrane-bound TGF-β and OX40. J Innate Immun (2016) 8(1):30–42. doi: 10.1159/000431150 26067079 PMC6738770

[106] Langhans B Alwan AW Krämer B Glässner A Lutz P Strassburg CP. Regulatory CD4+ T cells modulate the interaction between NK cells and hepatic stellate cells by acting on either cell type. J Hepatol (2015) 62(2):398–404. doi: 10.1016/j.jhep.2014.08.038 25195554

[107] Sarhan D Hippen KL Lemire A Hying S Luo X Lenvik T. Adaptive NK cells resist regulatory T-cell suppression driven by IL37. Cancer Immunol Res (2018) 6(7):766–75. doi: 10.1158/2326-6066.CIR-17-0498 PMC6030483 29784636

[108] Sitrin J Ring A Garcia KC Benoist C Mathis D. Regulatory T cells control NK cells in an insulitic lesion by depriving them of IL-2. J Exp Med (2013) 210(6):1153–65. doi: 10.1084/jem.20122248 PMC3674700 23650440

[109] Littwitz-Salomon E Malyshkina A Schimmer S Dittmer U. The cytotoxic activity of natural killer cells is suppressed by IL-10 regulatory T cells during acute retroviral infection. Front Immunol (2018) 9:1947. doi: 10.3389/fimmu.2018.01947 30210499 PMC6119693

[110] Hirohashi T Chase CM DellaPelle P Sebastian D Farkesh E Colvin RB. Depletion of T regulatory cells promotes natural killer cell-mediated cardiac allograft vasculopathy. Transplantation (2014) 98(8):828–34. doi: 10.1097/TP.0000000000000329 PMC4203423 25321164

[111] Mahr B Pilat N Maschke S Granofszky N Schwarz C Unger L. Regulatory T cells promote natural killer cell education in mixed chimeras. Am J Transplant (2017) 17(12):3049–59. doi: 10.1111/ajt.14342 28489338

[112] Jeffery HC Braitch MK Bagnall C Hodson J Jeffery LE Wawman RE. Changes in natural killer cells and exhausted memory regulatory T Cells with corticosteroid therapy in acute autoimmune hepatitis. Hepatol Commun (2018) 2(4):421–36. doi: 10.1002/hep4.1163 PMC5880196 29619420

[113] Tsuda S Nakashima A Shima T Saito S. New paradigm in the role of regulatory T cells during pregnancy. Front Immunol (2019) 10:573. doi: 10.3389/fimmu.2019.00573 30972068 PMC6443934

[114] Liu M Liang S Zhang C. NK cells in autoimmune diseases: protective or pathogenic? Front Immunol (2021) 12:624687. doi: 10.3389/fimmu.2021.624687 33777006 PMC7994264

[115] Vandenhaute J Wouters CH Matthys P. Natural killer cells in systemic autoinflammatory diseases: A focus on systemic juvenile idiopathic arthritis and macrophage activation syndrome. Front Immunol (2019) 10:3089. doi: 10.3389/fimmu.2019.03089 32010140 PMC6974473

[116] Migita K Fujita Y Asano T Sato S. The expanding spectrum of autoinflammatory diseases. Intern Med (2023) 62(1):43–50. doi: 10.2169/internalmedicine.9279-21 PMC9876706 36596474

[117] Bennstein SB. Unraveling natural killer T-cells development. Front Immunol (2017) 8:1950. doi: 10.3389/fimmu.2017.01950 29375573 PMC5767218

[118] Azuma T Takahashi T Kunisato A Kitamura T Hirai H. Human CD4+ CD25+ regulatory T cells suppress NKT cell functions. Cancer Res (2003) 63(15):4516–20. 12907625

[119] Venken K Decruy T Aspeslagh S Van Calenbergh S Lambrecht BN Elewaut D. Bacterial CD1d-restricted glycolipids induce IL-10 production by human regulatory T cells upon cross-talk with invariant NKT cells. J Immunol (2013) 191(5):2174–83. doi: 10.4049/jimmunol.1300562 23898038

[120] Hua J Liang S Ma X Webb TJ Potter JP Li Z. The interaction between regulatory T cells and NKT cells in the liver: a CD1d bridge links innate and adaptive immunity. PloS One (2011) 6(11):e27038. doi: 10.1371/journal.pone.0027038 22073248 PMC3206882

[121] Ihara F Sakurai D Takami M Kamata T Kunii N Yamasaki K. Regulatory T cells induce CD4 NKT cell anergy and suppress NKT cell cytotoxic function. Cancer Immunol Immunother (2019) 68(12):1935–47. doi: 10.1007/s00262-019-02417-6 PMC11028189 31641795

[122] Nguyen KD Vanichsarn C Nadeau KC. Increased cytotoxicity of CD4+ invariant NKT cells against CD4+CD25hiCD127lo/- regulatory T cells in allergic asthma. Eur J Immunol (2008) 38(7):2034–45. doi: 10.1002/eji.200738082 PMC4217522 18581330

[123] Li L Tu J Jiang Y Zhou J Schust DJ. Regulatory T cells decrease invariant natural killer T cell-mediated pregnancy loss in mice. Mucosal Immunol (2017) 10(3):613–23. doi: 10.1038/mi.2016.84 27706127

[124] Hongo D Tang X Dutt S Nador RG Strober S. Interactions between NKT cells and Tregs are required for tolerance to combined bone marrow and organ transplants. Blood (2012) 119(6):1581–9. doi: 10.1182/blood-2011-08-371948 PMC3286219 22174155

[125] La Cava A Van Kaer L Fu Dong S. CD4+CD25+ Tregs and NKT cells: regulators regulating regulators. Trends Immunol (2006) 27(7):322–7. doi: 10.1016/j.it.2006.05.003 16735139

[126] Berzins SP Ritchie DS. Natural killer T cells: drivers or passengers in preventing human disease? Nat Rev Immunol (2014) 14(9):640–6. doi: 10.1038/nri3725 25103356

[127] Dong R Zhang Y Xiao H Zeng X. Engineering γδ T cells: recognizing and activating on their own way. Front Immunol (2022) 13:889051. doi: 10.3389/fimmu.2022.889051 35603176 PMC9120431

[128] Piepke M Clausen BH Ludewig P Vienhues JH Bedke T Javidi E. Interleukin-10 improves stroke outcome by controlling the detrimental Interleukin-17A response. J Neuroinflammation (2021) 18(1):265. doi: 10.1186/s12974-021-02316-7 34772416 PMC8590298

[129] Park S-G Mathur R Long M Hosh N Hao L Hayden MS. T regulatory cells maintain intestinal homeostasis by suppressing γδ T cells. Immunity (2010) 33(5):791–803. doi: 10.1016/j.immuni.2010.10.014 21074460 PMC2996054

[130] Li L Wu C-Y. CD4+ CD25+ Treg cells inhibit human memory gammadelta T cells to produce IFN-gamma in response to M tuberculosis antigen ESAT-6. Blood (2008) 111(12):5629–36. doi: 10.1182/blood-2008-02-139899 18388182

[131] Yurchenko E Levings MK Piccirillo CA. CD4+ Foxp3+ regulatory T cells suppress γδ T-cell effector functions in a model of T-cell-induced mucosal inflammation. Eur J Immunol (2011) 41(12):3455–66. doi: 10.1002/eji.201141814 21956668

[132] Yi Y He H-W Wang J-X Cai X-Y Li Y-W Zhou J. The functional impairment of HCC-infiltrating γδ T cells, partially mediated by regulatory T cells in a TGFβ- and IL-10-dependent manner. J Hepatol (2013) 58(5):977–83. doi: 10.1016/j.jhep.2012.12.015 23262246

[133] Blanco-Domínguez R de la Fuente H Rodríguez C Martín-Aguado L Sánchez-Díaz R Jiménez-Alejandre R. CD69 expression on regulatory T cells protects from immune damage after myocardial infarction. J Clin Invest (2022) 132(21):e152418. doi: 10.1093/eurheartj/ehab724.3239 36066993 PMC9621142

[134] Faustino LD Griffith JW Rahimi RA Nepal K Hamilos DL Cho JL. Interleukin-33 activates regulatory T cells to suppress innate γδ T cell responses in the lung. Nat Immunol (2020) 21(11):1371–83. doi: 10.1038/s41590-020-0785-3 PMC7578082 32989331

[135] Petermann F Rothhammer V Claussen MC Haas JD Blanco LR Heink S. γδ T cells enhance autoimmunity by restraining regulatory T cell responses via an interleukin-23-dependent mechanism. Immunity (2010) 33(3):351–63. doi: 10.1016/j.immuni.2010.08.013 PMC3008772 20832339

[136] Visperas A Shen B Min B. γδ T cells restrain extrathymic development of Foxp3+-inducible regulatory T cells via IFN-γ. Eur J Immunol (2014) 44(8):2448–56. doi: 10.1002/eji.201344331 PMC4141022 24799116

[137] Liu W Moussawi M Roberts B Boyson JE Huber SA. Cross-regulation of T regulatory-cell response after coxsackievirus B3 infection by NKT and γδ T cells in the mouse. Am J Pathol (2013) 183(2):441–9. doi: 10.1016/j.ajpath.2013.04.015 PMC3730787 23746656

[138] Huber SA. Depletion of gammadelta+ T cells increases CD4+ FoxP3 (T regulatory) cell response in coxsackievirus B3-induced myocarditis. Immunology (2009) 127(4):567–76. doi: 10.1111/j.1365-2567.2008.03034.x PMC2729534 19604307

[139] Peters C Kabelitz D Wesch D. Regulatory functions of γδ T cells. Cell Mol Life Sci (2018) 75(12):2125–35. doi: 10.1007/s00018-018-2788-x PMC11105251 29520421

[140] Hazenberg MD Spits H. Human innate lymphoid cells. Blood (2014) 124(5):700–9. doi: 10.1182/blood-2013-11-427781 24778151

[141] Tait Wojno ED Artis D. Emerging concepts and future challenges in innate lymphoid cell biology. J Exp Med (2016) 213(11):2229–48. doi: 10.1084/jem.20160525 PMC5068238 27811053

[142] Rigas D Lewis G Aron JL Wang B Banie H Sankaranarayanan I. Type 2 innate lymphoid cell suppression by regulatory T cells attenuates airway hyperreactivity and requires inducible T-cell costimulator-inducible T-cell costimulator ligand interaction. J Allergy Clin Immunol (2017) 139(5):1468–1477.e2. doi: 10.1016/j.jaci.2016.08.034 PMC5378695 27717665

[143] Khumalo J Kirstein F Hadebe S Brombacher F. IL-4Rα signaling in CD4+CD25+FoxP3+ T regulatory cells restrains airway inflammation via limiting local tissue IL-33. JCI Insight (2020) 5(20):e136206. doi: 10.1172/jci.insight.136206 32931477 PMC7605533

[144] Krishnamoorthy N Burkett PR Dalli J Abdulnour R-EE Colas R Ramon S. Cutting edge: maresin-1 engages regulatory T cells to limit type 2 innate lymphoid cell activation and promote resolution of lung inflammation. J Immunol (2015) 194(3):863–7. doi: 10.4049/jimmunol.1402534 PMC4297713 25539814

[145] Liu W Zeng Q Wen Y Tang Y Yan S Li Y. Inhibited interleukin 35 expression and interleukin 35-induced regulatory T cells promote type II innate lymphoid cell response in allergic rhinitis. Ann Allergy Asthma Immunol (2021) 126(2)152–161.e1. doi: 10.1016/j.anai.2020.08.005 32771356

[146] Fan X Xu Z-B Li C-L Zhang H-Y Peng Y-Q He B-X. Mesenchymal stem cells regulate type 2 innate lymphoid cells via regulatory T cells through ICOS-ICOSL interaction. Stem Cells (2021) 39(7):975–87. doi: 10.1002/stem.3369 PMC8360040 33662168

[147] Glaubitz J Wilden A Golchert J Homuth G Völker U Bröker BM. In mouse chronic pancreatitis CD25FOXP3 regulatory T cells control pancreatic fibrosis by suppression of the type 2 immune response. Nat Commun (2022) 13(1):4502. doi: 10.1038/s41467-022-32195-2 35922425 PMC9349313

[148] Gao X Lin J Zheng Y Liu S Liu C Liu T. Type 2 innate lymphoid cells regulation by regulatory T cells attenuates atherosclerosis. J Mol Cell Cardiol (2020) 145:99–111. doi: 10.1016/j.yjmcc.2020.05.017 32526223

[149] Van Gool F Molofsky AB Morar MM Rosenzwajg M Liang H-E Klatzmann D. Interleukin-5-producing group 2 innate lymphoid cells control eosinophilia induced by interleukin-2 therapy. Blood (2014) 124(24):3572–6. doi: 10.1182/blood-2014-07-587493 PMC4256909 25323825

[150] Zhou L Chu C Teng F Bessman NJ Goc J Santosa EK. Innate lymphoid cells support regulatory T cells in the intestine through interleukin-2. Nature (2019) 568(7752):405–9. doi: 10.1038/s41586-019-1082-x PMC6481643 30944470

[151] Roediger B Kyle R Tay SS Mitchell AJ Bolton HA Guy TV. IL-2 is a critical regulator of group 2 innate lymphoid cell function during pulmonary inflammation. J Allergy Clin Immunol (2015) 136(6):1653–1663.e7. doi: 10.1016/j.jaci.2015.03.043 26025126

[152] Savic S Caseley EA McDermott MF. Moving towards a systems-based classification of innate immune-mediated diseases. Nat Rev Rheumatol (2020) 16(4):222–37. doi: 10.1038/s41584-020-0377-5 32107482

[153] Gonzalez LL Garrie K Turner MD. Type 2 diabetes - An autoinflammatory disease driven by metabolic stress. Biochim Biophys Acta Mol Basis Dis (2018) 1864(11):3805–23. doi: 10.1016/j.bbadis.2018.08.034 30251697

[154] Zhang J Ja C Gao C Sun X Wang L Hu Z. Maggot treatment promotes healing of diabetic foot ulcer wounds possibly by upregulating Treg levels. Diabetes Res Clin Pract (2022) 184:109187. doi: 10.1016/j.diabres.2021.109187 35016990

[155] Guo F Ren Z Liu D Wang L Hou X Chen W. The inhibitory effect of regulatory T cells on the intimal hyperplasia of tissue-engineered blood vessels in diabetic pigs. Front Bioeng Biotechnol (2022) 10:929867. doi: 10.3389/fbioe.2022.929867 35957644 PMC9360552

[156] Deliyanti D Talia DM Zhu T Maxwell MJ Agrotis A Jerome JR. Foxp3+ Tregs are recruited to the retina to repair pathological angiogenesis. Nat Commun (2017) 8(1):748. doi: 10.1038/s41467-017-00751-w 28963474 PMC5622066

[157] Eller K Kirsch A Wolf AM Sopper S Tagwerker A Stanzl U. Potential role of regulatory T cells in reversing obesity-linked insulin resistance and diabetic nephropathy. Diabetes (2011) 60(11):2954–62. doi: 10.2337/db11-0358 PMC3198056 21911743

[158] Sabapathy V Stremska ME Mohammad S Corey RL Sharma PR Sharma R. Novel immunomodulatory cytokine regulates inflammation, diabetes, and obesity to protect from diabetic nephropathy. Front Pharmacol (2019) 10:572. doi: 10.3389/fphar.2019.00572 31191312 PMC6540785

[159] Zhu L Song H Zhang L Meng H. Characterization of IL-17-producing Treg cells in type 2 diabetes patients. Immunol Res (2019) 67(4-5):443–9. doi: 10.1007/s12026-019-09095-7 31713831

[160] Sheikh V Zamani A Mahabadi-Ashtiyani E Tarokhian H Borzouei S Alahgholi-Hajibehzad M. Decreased regulatory function of CD4+CD25+CD45RA+ T cells and impaired IL-2 signalling pathway in patients with type 2 diabetes mellitus. Scand J Immunol (2018) 88(4):e12711. doi: 10.1111/sji.12711 30270447

[161] de Ramon L Guiteras J Guiteras R Cruzado JM Grinyó JM Torras J. The costimulatory pathways and T regulatory cells in ischemia-reperfusion injury: A strong arm in the inflammatory response? Int J Mol Sci (2018) 19(5):1283. doi: 10.3390/ijms19051283 29693595 PMC5983665

[162] Wang M Thomson AW Yu F Hazra R Junagade A Hu X. Regulatory T lymphocytes as a therapy for ischemic stroke. Semin Immunopathol (2023) 45(3):329–46. doi: 10.1007/s00281-022-00975-z PMC10239790 36469056

[163] Feng M Wang Q Zhang F Lu L. Ex vivo induced regulatory T cells regulate inflammatory response of Kupffer cells by TGF-beta and attenuate liver ischemia reperfusion injury. Int Immunopharmacol (2012) 12(1):189–96. doi: 10.1016/j.intimp.2011.11.010 22155100

[164] Wang Y Wang C Shen L Xu D. The role of regulatory T cells in heart repair after myocardial infarction. J Cardiovasc Transl Res (2023) 16(3):590–7. doi: 10.1007/s12265-022-10290-5 37347425

[165] Xia N Lu Y Gu M Li N Liu M Jiao J. A unique population of regulatory T cells in heart potentiates cardiac protection from myocardial infarction. Circulation (2020) 142(20):1956–73. doi: 10.1161/CIRCULATIONAHA.120.046789 32985264

[166] Jun C Qingshu L Ke W Ping L Jun D Jie L. Protective effect of CXCR3^+^CD4^+^CD25^+^Foxp3^+^ Regulatory T cells in renal ischemia-reperfusion injury. Mediators Inflamm (2015) 2015:360973. doi: 10.1155/2015/360973 26273136 PMC4530276

[167] Ridker PM Everett BM Thuren T MacFadyen JG Chang WH Ballantyne C. Antiinflammatory therapy with canakinumab for atherosclerotic disease. N Engl J Med (2017) 377(12):1119–31. doi: 10.1056/NEJMoa1707914 28845751

[168] Tardif J-C Kouz S Waters DD Bertrand OF Diaz R Maggioni AP. Efficacy and safety of low-dose colchicine after myocardial infarction. N Engl J Med (2019) 381(26):2497–505. doi: 10.1056/NEJMoa1912388 31733140

[169] Nidorf SM Fiolet ATL Mosterd A Eikelboom JW Schut A Opstal TSJ. Colchicine in patients with chronic coronary disease. N Engl J Med (2020) 383(19):1838–47. doi: 10.1056/NEJMoa2021372 32865380

[170] George J Schwartzenberg S Medvedovsky D Jonas M Charach G Afek A. Regulatory T cells and IL-10 levels are reduced in patients with vulnerable coronary plaques. Atherosclerosis (2012) 222(2):519–23. doi: 10.1016/j.atherosclerosis.2012.03.016 22575708

[171] Olson NC Sitlani CM Doyle MF Huber SA Landay AL Tracy RP. Innate and adaptive immune cell subsets as risk factors for coronary heart disease in two population-based cohorts. Atherosclerosis (2020) 300:47–53. doi: 10.1016/j.atherosclerosis.2020.03.011 32209232 PMC7276206

[172] Mor A Planer D Luboshits G Afek A Metzger S Chajek-Shaul T. Role of naturally occurring CD4+ CD25+ regulatory T cells in experimental atherosclerosis. Arterioscler Thromb Vasc Biol (2007) 27(4):893–900. doi: 10.1161/01.ATV.0000259365.31469.89 17272749

[173] Winkels H Meiler S Lievens D Engel D Spitz C Bürger C. CD27 co-stimulation increases the abundance of regulatory T cells and reduces atherosclerosis in hyperlipidaemic mice. Eur Heart J (2017) 38(48):3590–9. doi: 10.1093/eurheartj/ehx517 29045618

[174] Butcher MJ Filipowicz AR Waseem TC McGary CM Crow KJ Magilnick N. Atherosclerosis-driven treg plasticity results in formation of a dysfunctional subset of plastic IFNγ+ Th1/tregs. Circ Res (2016) 119(11):1190–203. doi: 10.1161/CIRCRESAHA.116.309764 PMC5242312 27635087

[175] Li J McArdle S Gholami A Kimura T Wolf D Gerhardt T. CCR5+T-bet+FoxP3+ Effector CD4 T cells drive atherosclerosis. Circ Res (2016) 118(10):1540–52. doi: 10.1161/CIRCRESAHA.116.308648 PMC4867125 27021296

[176] Ali AJ Makings J Ley K. Regulatory T cell stability and plasticity in atherosclerosis. Cells (2020) 9(12):2665. doi: 10.3390/cells9122665 33322482 PMC7764358

